# SAM3TGNet: A SAM3 Feature Encoding and Global Context Spatiotemporal Attention-Enhanced Change Detection Method for Optical Remote Sensing Images

**DOI:** 10.3390/s26175469

**Published:** 2026-08-29

**Authors:** Jiayin Zhang, Nan Mo, Gege Ma, Bangyan Tang

**Affiliations:** School of Geomatics Science and Technology, Nanjing Tech University, Nanjing 211816, China; zhangjy@njtech.edu.cn (J.Z.); magege@njtech.edu.cn (G.M.);

**Keywords:** change detection, remote sensing image, segment anything model 3 (SAM3) feature encoding, global context spatial attention, dynamic weighted loss, feature fusion

## Abstract

Change detection in high-resolution optical remote sensing images is essential for dynamic surface monitoring and land resource management. However, existing methods still face challenges in representing multiscale change information, accurately locating change boundaries, and handling the imbalance between changed and unchanged samples. This study proposes SAM3TGNet, which transforms the general visual features of Segment Anything Model 3 (SAM3) into change-oriented representations through lightweight channel adaptation, multiscale cross-temporal interaction, and spatiotemporal feature fusion, while the Global Context Spatial Attention (GCSA) module and dynamic weighted loss enhance boundary representation and alleviate class imbalance, respectively. First, a SAM3-based bi-temporal remote sensing image feature encoder with channel adapters is developed, where intermediate multiscale feature layers are exploited to enhance semantic representation and generalization for diverse change patterns. Second, the GCSA module is introduced after spatiotemporal feature fusion to model global dependencies and enhance local details, improving boundary localization accuracy. Third, a dynamic weighted change loss function is designed to adaptively adjust the contribution of changed pixels according to their proportion in each batch, reducing background bias caused by sample imbalance and improving change localization and completeness. Experiments on the Wuhan University Change Detection (WHU-CD) and Sun Yat-sen University Change Detection (SYSU-CD) datasets demonstrate that the proposed method achieves F1-scores of 94.08 ± 0.12% and 82.91 ± 0.20%, respectively, outperforming existing approaches in multiscale change detection and boundary delineation.

## 1. Introduction

Change detection in high-resolution optical remote sensing images aims to identify changed regions of land cover or ground objects by comparing remote sensing images of the same area acquired at different times, and it is an important research direction in remote sensing image interpretation and dynamic surface monitoring. With the continuous development of Earth observation technologies and sensors, remote sensing images have been remarkably improved in spatial resolution and texture details, making change detection valuable in various applications, such as urban planning [1], land-use updating [2], and disaster damage assessment [3].

However, while high-resolution remote sensing images provide rich spatial details, they also introduce challenges such as complex background textures, considerable scale differences among changed regions, and interference from shadows and illumination variations. These factors often lead to missed detections, false detections, and inaccurate boundary localization of changed regions [4]. Before automated methods became widely used, changes in remote sensing images were commonly identified through manual visual interpretation, which was inefficient and dependent on operator experience [5]. Early change detection methods mainly relied on handcrafted features. For example, change vector analysis based on image transformation [6] determines changed regions by calculating the differences between multitemporal images in the spectral space. In addition, unsupervised methods combining principal component analysis and K-means clustering have also been used to distinguish changed and unchanged regions without labeled samples [7]. With the development of machine learning, classifier-based methods have gradually become an important branch of traditional remote sensing image change detection. Such methods typically begin by extracting spectral, textural, shape, or object-level statistical features from bi-temporal remote sensing images, and then feed these manually designed features into shallow classifiers such as decision trees [8], random forests [9], and support vector machines [10], thereby transforming change detection into a binary classification problem between changed and unchanged classes. Compared to simple threshold segmentation methods, machine learning methods can utilize training samples to learn more complex class discrimination boundaries of changes and unchanges, thereby improving the ability to identify changed regions to a certain extent [11]; however, their performance still depends on feature design, sample quality, and classifier parameter settings, and their generalization ability is limited in complex and high-resolution scenes.

In recent years, with the development of deep learning methods [12], change detection in remote sensing imagery has gradually shifted from traditional methods to learning-based approaches utilizing typical deep network architectures such as Convolutional Neural Networks (CNN) and Transformers. Unlike traditional methods that rely on handcrafted features or predefined change measures, deep learning-based change detection methods automatically extract multi-layer semantic features from bi-temporal images through an encoder and then characterize the change information between the paired images using techniques such as feature concatenation, feature differencing, or feature fusion [13,14]. Finally, a decoder generates pixel-level change detection results. Daudt et al. [13] proposed three fully convolutional change detection architectures, FC-EF, FC-Siam-conc, and FC-Siam-diff, which fuse bi-temporal information through input-level early fusion, feature concatenation, and feature differencing, respectively. Subsequently, Fang et al. [14] proposed SNUNet-CD, which introduces dense multilevel feature connections into a Siamese network to enhance feature aggregation across different levels and reduce the loss of localization information. However, CNN-based methods mainly rely on local convolutional operations for feature extraction. Although they can effectively capture local information such as textures and boundaries, their ability to model large-scale spatial context and long-range cross-temporal semantic relationships remains limited. To address this limitation, Chen et al. [15] proposed the BIT method, which maps bi-temporal image features into compact high-level semantic representations and employs the Transformer encoder to model the spatiotemporal contextual relationships between bi-temporal images. This design enhances the model’s ability to capture long-range dependencies and global semantic information, thereby alleviating the insufficient contextual aggregation in change detection maps to some extent.

Although deep learning-based methods have achieved considerable progress over traditional change detection methods, they still face several challenges. First, changed regions usually exhibit large variations in scale, shape, and spatial distribution, while conventional feature encoding networks still have limited semantic representation capability for multiscale changed regions. Second, the boundaries of changed regions are usually influenced by the shape of objects and background environmental factors. Traditional attention mechanisms may therefore lead to blurred boundaries, local discontinuities, or false detections caused by pseudo-changes. Finally, in change detection tasks, changed pixels are usually far fewer than unchanged pixels. The imbalance between positive and negative samples may bias the training process toward unchanged regions, thereby increasing interference in changed region localization and affecting the change detection accuracy. Therefore, how to simultaneously enhance multiscale semantic representation, the localization accuracy and completeness of changed regions, and changed-region learning under class-imbalanced conditions remains a key issue in high-resolution remote sensing image change detection.

To further address the above problems, the development of vision foundation models and attention mechanisms provides new ideas for remote sensing change detection. Vision foundation models represented by the Segment Anything Model (SAM) series [16] are pretrained on large-scale visual data and possess strong general visual representation capability and object-structure perception ability. In this study, we propose a change detection method called SAM3TGNet, which introduces SAM3 [17] as a visual feature encoder for remote sensing image change detection. By leveraging its advantages in object-structure perception and region representation, the proposed method enhances the representation capability for complex ground-object structures and multiscale changed regions. However, SAM3 is not specifically designed for remote sensing image change detection and cannot explicitly model differences between bitemporal images. Compared with the original SAM3, SAM3TGNet freezes the SAM3 encoder during training and introduces lightweight channel mappings to adapt its multiscale outputs to the dimensional requirements of the subsequent change detection modules. Cross-temporal interaction is then employed to explicitly model the relationships between the paired bitemporal features, while spatiotemporal fusion integrates spatial change cues with temporal evolution information, thereby transforming the general visual features of SAM3 into change-specific representations. Nevertheless, complex backgrounds, shadow occlusions, and pseudo-change regions may still interfere with the fused change features, resulting in local discontinuities within changed regions, missed detections of small changed objects, and inaccurate boundary localization. To address this issue, the Global Context Spatial Attention (GCSA) mechanism [18] is further introduced based on SAM3 feature encoding. By modeling global contextual relationships and local spatial details, GCSA further enhances the change features after spatiotemporal feature fusion. On the one hand, GCSA enhances the consistency of feature responses within the same changed region, thereby alleviating local discontinuities in changed areas; on the other hand, it strengthens boundary and fine-structure information, thereby improving the boundary localization accuracy of changed regions. In addition, a dynamic weighted loss function is designed to adaptively adjust the positive sample weight according to the proportion of changed pixels in each batch, alleviating the class imbalance caused by the sparsity of changed-region samples.

Based on the above analysis, this study aims to improve the representation of multiscale change information and the localization accuracy of change boundaries in high-resolution optical remote sensing images, while reducing the influence of class imbalance on changed-region detection. To this end, a unified change detection framework is constructed by integrating SAM3-based bi-temporal feature encoding, cross-temporal interaction and spatiotemporal feature fusion, GCSA-based feature enhancement, and dynamic weighted supervision. The main contributions are as follows:

(1) A bitemporal multilevel feature adaptation scheme based on a frozen SAM3 encoder is constructed. The scheme selects the last three high-level semantic feature maps from the SAM3 encoder and connects them to multiscale bitemporal change modeling through lightweight channel mappings. It uses the multiscale semantic and object-structure priors of SAM3 without updating its parameters.
(2) The GCSA mechanism [18] is applied to multiscale change features after feature interaction and fusion. At the low and middle scales, cross-temporal spatial change features are fused with temporal evolution features. At the largest semantic scale, the spatial change features are retained. GCSA is then applied at all three scales to improve intra-region consistency and boundary localization.
(3) A dynamic weighted loss function is constructed to adaptively adjust the changed-class weight according to the proportion of changed pixels in each batch. Specifically, the proposed loss combines dynamically weighted binary cross-entropy (BCE) loss [19] with a loss based on the Dice similarity coefficient (Dice loss) [20] and is applied to the final prediction and multiscale auxiliary predictions. This loss function enhances the contribution of sparse changed pixels during loss optimization and alleviates the background bias caused by the imbalance between positive and negative samples, thereby further improving the model’s recognition capability for changed regions.

The rest of this article is organized as follows: Section 2 reviews the research status relevant to remote sensing image change detection. Section 3 describes the proposed SAM3TGNet network architecture and its module design in detail. Section 4 presents the experimental datasets, evaluation metrics, comparative experiments, and ablation results. Finally, Section 5 concludes this study and discusses future research directions.

## 2. Related Work

Considering the problems of missed detection of multiscale changed regions, pseudo-change interference, and inaccurate boundary localization in high-resolution remote sensing image change detection, recent studies have mainly focused on two aspects: attention mechanism enhancement and the transfer of vision foundation models. The former enhances the model’s ability to focus on key changed regions by adaptively modeling channel, spatial, spatiotemporal, or global contextual information. The latter leverages the general representation capability and object-structure priors of large-scale pretrained visual models to improve the representation of complex ground-object structures and multiscale changed regions.

### 2.1. Attention Mechanism Enhancement

With the increasing complexity of high-resolution remote sensing scenes, change detection methods need not only to extract semantic differences between bitemporal images, but also to highlight real changed regions and suppress pseudo-change interference in complex backgrounds. To enhance the capability of models to extract critical change information, attention mechanisms have gradually been introduced into remote sensing image change detection tasks. According to the modeling objectives, existing attention mechanisms can generally be categorized into channel attention, spatial attention, channel–spatial hybrid attention, spatiotemporal attention, and hierarchical attention mechanisms.

Early attention-based methods mainly enhance changed-region responses through feature reweighting along the channel or spatial dimensions. Song et al. [21] proposed the AGCDetNet method, which introduces spatial attention and channel attention into the building change detection task. By using attention guidance to enhance the discrimination between changed objects and backgrounds, this method improves the detection performance of building changed regions in high-resolution images. This method demonstrates the effectiveness of channel and spatial attention in enhancing changed targets. However, its ability to model complex ground-object types and large-scale spatial contextual relationships remains limited. Lei et al. [22] designed the USSFCNet method, which learns three-dimensional spatial–spectral attention weights through a spatial–spectral feature collaboration mechanism, thereby enhancing changed-region feature representation while maintaining a lightweight model structure. This method has advantages in lightweight design and spatial–spectral collaboration, but it remains insufficient in jointly modeling global contextual relationships and local boundary details within fused features.

To enhance the capability of modeling bitemporal change relationships and multiscale contextual representation, some methods have further introduced spatiotemporal attention and hierarchical attention based on channel and spatial attention. Chen et al. [23] developed the STANet method, which introduces a spatial–temporal attention mechanism into a Siamese network. By modeling the associations among different spatial positions and temporal relationships in bitemporal images, this method improves the recognition capability for real changed regions under illumination variations and registration errors. This method enhances the modeling of bitemporal spatial relationships. However, its attention mechanism is mainly used to strengthen the associations between bitemporal features, and its constraints on change boundary details and small-scale target structures remain insufficient. Han et al. [24] proposed the HANet method, which fuses contextual features from different levels through a hierarchical attention module and combines it with a foreground-balanced sampling strategy to alleviate the imbalance between changed and unchanged pixels. This method improves multiscale feature representation and alleviates class imbalance. However, its attention mechanism mainly operates on hierarchical feature fusion, and it remains insufficient in synergistically modeling global spatial continuity and local boundary details in complex scenes.

Overall, existing attention mechanisms have evolved from channel attention and spatial attention to spatiotemporal attention and hierarchical attention, enabling models to enhance their focus on changed regions from different perspectives. Nevertheless, these methods remain insufficient in jointly enhancing global spatial consistency and local boundary details within fused features. To address this issue, this study introduces the GCSA mechanism module to act on multiscale features after spatiotemporal fusion. Unlike attention methods that focus only on channel, spatial, or hierarchical features, GCSA places greater emphasis on further enhancing spatiotemporally fused features. On the one hand, it enhances the consistency of feature responses within the same changed region through global contextual modeling, making changed regions more continuous and complete. On the other hand, it supplements local spatial details to strengthen change boundaries and fine structural information, thereby improving inaccurate boundary localization and false detection caused by pseudo-changes.

### 2.2. Vision Foundation Models

In recent years, vision foundation models have been pretrained on large-scale visual data and have demonstrated strong general feature representation and knowledge transfer capabilities, providing a new research direction for remote sensing image change detection. Vision foundation models can learn richer visual priors and semantic knowledge from large-scale image, image–text, or self-supervised data, thereby alleviating, to some extent, the problems of limited annotated samples, large scene variations, and insufficient model generalization in remote sensing change detection.

In early studies on transferring vision foundation models to remote sensing change detection, Vision-language models represented by CLIP have attracted considerable attention [25]. Dong et al. [26] proposed the ChangeCLIP framework, which introduces the vision–language representation capability of CLIP into remote sensing image change detection. By using textual semantic information to assist the model in understanding high-level change semantics in bitemporal images, this method enhances the discriminative capability for changed regions. Li et al. [27] developed the BAN method, which freezes CLIP and designs bitemporal adaptation branches and a bridging module, improving the model’s change detection capability under limited-sample conditions. However, the above methods still have room for improvement in spatially representing complex ground-object structures, change boundaries, and multiscale objects.

In addition to vision–language models, self-supervised vision foundation models have also gradually been applied to remote sensing change detection tasks. The DINO family of models [28] obtain strong visual representation capabilities through self-supervised learning and can learn visual features without requiring a large amount of manually annotated data. Dong et al. [29] designed the PeftCD method, which introduces vision foundation models such as DINOv3 and SAM2 into the remote sensing change detection framework to alleviate pseudo-change interference, annotation scarcity, and cross-domain generalization difficulties in multitemporal remote sensing images. Cheng et al. [30] further proposed the ChangeDINO method, which uses semantic and contextual features provided by frozen DINOv3 to construct a multiscale Siamese change detection network, helping to improve the model’s generalization capability under small-sample and complex imaging conditions. However, these models mainly focus on general visual feature learning. When directly transferred to pixel-level remote sensing image change detection, they may still lack in representing local boundary details, be less robust in detecting small changed targets, and have difficulty fully accommodating the spatial structures of complex changed regions.

To address the insufficient spatial detail representation of vision foundation models in remote sensing change detection, subsequent studies have introduced vision foundation models with segmentation priors. Ding et al. [31] proposed the SAM-CD, which introduces the visual encoding capability of FastSAM into high-resolution remote sensing change detection, improving change detection accuracy while reducing sample dependency. Gao et al. [32] proposed the Meta-CD, which combines FastSAM with an additional CNN adaptation module, enabling the model to perform change detection with limited remote sensing training samples and improving its localization capability for changed regions. However, these methods mainly use vision foundation models as auxiliary feature extractors or task adaptation tools, and their utilization of the multiscale encoding capability of foundation models remains insufficient. As a result, missed detections or incomplete boundaries may still occur in changed regions with large differences, irregular shapes, and significant scale variations.

To improve the adaptability of vision foundation models to remote sensing images, Zhao et al. [33] developed the SAM-UNet, which combines SAM with the U-Net architecture and adjusts the receptive field through center scaling, thereby enhancing changed-region detection capability. Zhang et al. [34] proposed the SFCD-Net, which adapts SAM and incorporates a feature interaction module to reduce the domain discrepancy between natural images and remote sensing images, thereby alleviating missed detections of changed regions. Wei et al. [35] proposed the ASS-CD, which transfers pretrained visual models such as FastSAM and Swin Transformer to remote sensing change detection tasks through hierarchical adapters. Recent studies have further expanded the adaptation strategies of vision foundation models for remote sensing change detection. Parameter-efficient multiscale adaptation and low-rank exchange adaptation have been used to improve multiscale change representation and cross-domain generalization [36,37], while multi-source foundation model fusion and modality-bridged representation learning have further expanded change representation under pseudo-change interference and heterogeneous sensor conditions [38,39]. Collectively, these methods improve the adaptability of vision foundation models to remote sensing change detection through receptive-field adjustment, feature interaction, hierarchical or low-rank adaptation, and multi-source or cross-modal feature fusion [33,34,35,36,37,38,39]. However, owing to the domain gap between natural and remote sensing images, transferring vision foundation models to remote sensing tasks still requires task-specific fine-tuning or adaptation designs [40]. Moreover, despite the progress achieved by these methods, their capabilities for multiscale spatial feature encoding, complex ground-object contour representation, and boundary refinement can be further strengthened. Changed regions often exhibit large variations in scale and spatial distribution, whereas spatial details and semantic information from different feature levels are not always sufficiently coordinated, which limits the consistent representation of change patterns across scales. In addition, fine structural cues may be attenuated during feature adaptation and interaction, making irregular ground-object contours difficult to preserve; interference from complex backgrounds, shadows, and pseudo-change regions may further lead to fragmented responses within changed regions and local boundary deviations.

To address the above issues, this study introduces the SAM3 as the visual feature encoder for bitemporal remote sensing images and freezes its parameters to preserve the general visual representation capability acquired from large-scale pretraining. Compared with CLIP-like models that emphasize semantic alignment and DINO-like models that focus on general feature learning, SAM3 is more suitable for providing spatial visual priors for change detection, such as object structures, region contours, and boundary information. This study directly employs SAM3 to extract multiscale spatial features from bitemporal remote sensing images and maps them to the feature dimensions required by subsequent change detection modules through channel adapters. This design reduces feature dimension mismatch during the transfer of SAM3, compensates for the limitations of conventional change detection encoders and some vision foundation models in representing complex ground-object structures, multiscale changed regions, and boundary regions, and provides richer and more stable spatial semantic features for change discrimination. Building on the aforementioned research, this study further examines how SAM3 multiscale features are organized within the dual-phase change detection process. The frozen SAM3 features first undergo channel mapping and cross-phase interaction. At the low and medium scales, these features are fused with time-evolutionary features. Subsequently, GCSA is applied across the three scales to enhance the global consistency of change features and the local boundary details. During the training phase, batch-adaptive positive-class weighting is employed to mitigate background bias caused by the sparsity of changing pixels. Thus, the main contribution of this paper lies in the construction of a unified workflow that includes feature adaptation, interactive fusion, feature refinement, and class imbalance optimization.

## 3. Methodology

### 3.1. Network Architecture of the Proposed SAM3TGNet

**Figure 1:**
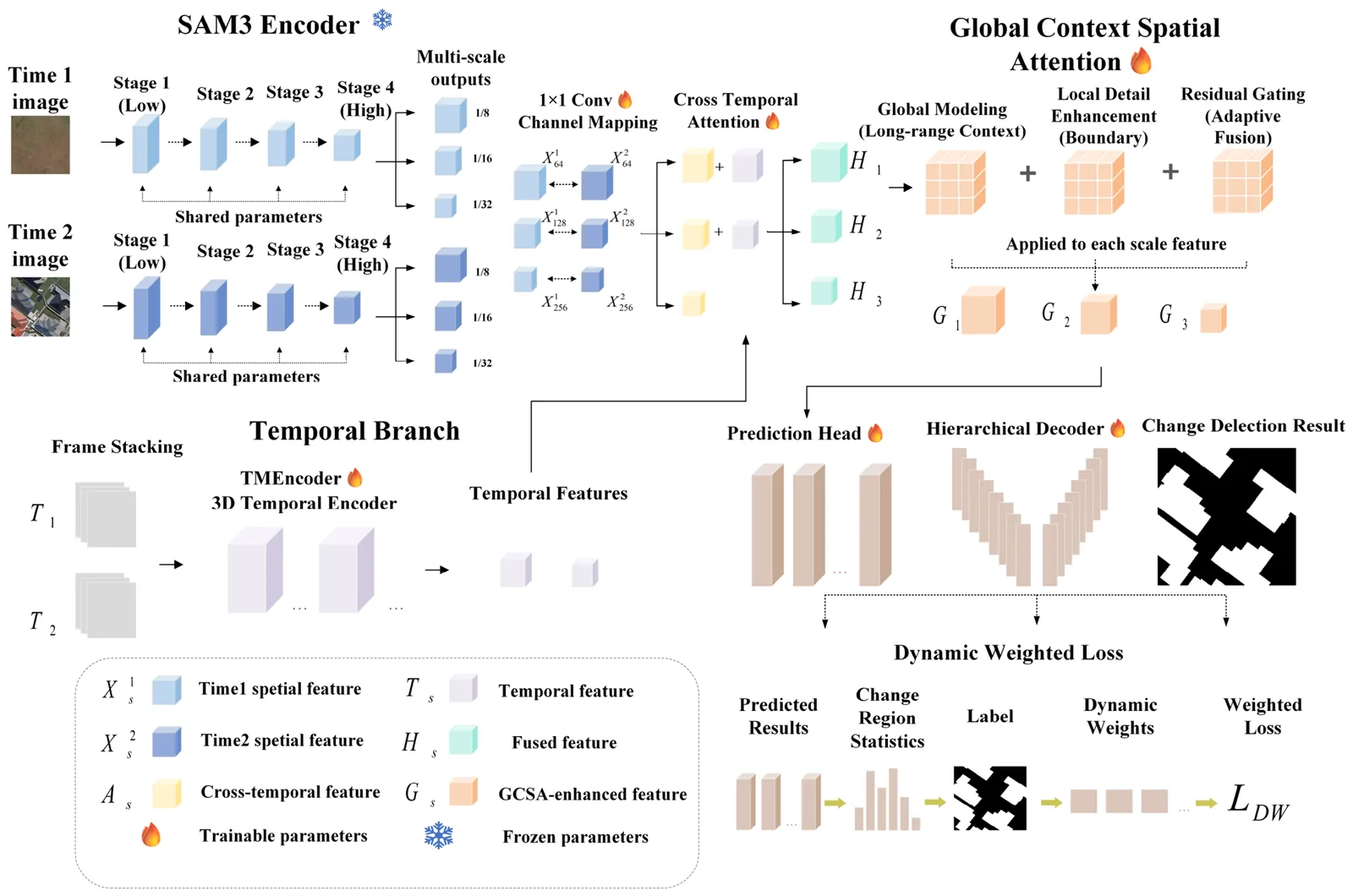
Overall framework of the proposed method. Light-blue, dark-blue, yellow, purple, cyan, and orange blocks denote Time 1 spatial features, Time 2 spatial features, cross-temporal features, temporal features, fused features, and GCSA-enhanced features, respectively. Beige blocks represent prediction, decoding, and loss-related components. The flame and snowflake symbols indicate trainable and frozen parameters, respectively.

This study proposes a change detection model named SAM3TGNet, which is based on SAM3 feature encoding and a global context spatial attention mechanism. Its overall framework is shown in Figure 1. The model takes bitemporal remote sensing images T1 and T2 as inputs and collaboratively models the change information between the two temporal images through a spatial branch and a temporal branch. The overall network mainly consists of three key components: SAM3-based multiscale feature encoding, GCSA-based spatiotemporal fusion feature enhancement, and a dynamic weighted loss function.

First, in the spatial branch, a SAM3 visual encoder with frozen parameters is employed to extract multiscale spatial features from the bitemporal remote sensing images separately. The SAM3 encoder generates hierarchical features at different scales through progressive feature extraction, and the last three feature levels with higher semantic representations are selected as multiscale spatial features, corresponding to 1/8, 1/16, and 1/32 of the input image resolution, respectively. Then these features are fed into the subsequent change detection modules through lightweight channel adaptation and cross-temporal feature interaction.

Second, in the temporal branch, the T1 and T2 images are linearly interpolated to construct an eight-frame pseudo-temporal sequence, which is fed into the 3D temporal encoder (TMEncoder) [41] to extract temporal evolution features. After temporal average and max pooling, the resulting high-level temporal feature is directly fused with the low-scale spatial change feature and bilinearly interpolated for alignment and fusion with the middle-scale spatial change feature. Since no corresponding temporal feature is available at the largest semantic scale, only the spatial change feature is retained at this scale. Then the GCSA mechanism [18] is introduced to further enhance the features at each scale. On the one hand, global contextual modeling aggregates spatial information and captures dependencies among channel subspaces, thereby improving the consistency and continuity of feature responses within the same changed region. On the other hand, the local detail enhancement branch supplements boundary and fine-structure information while suppressing interference from complex backgrounds and pseudo changes, thereby improving the localization capability and boundary representation ability of the model for complex changed regions.

Finally, during the training stage, a dynamic weighted loss function is adopted to optimize the model. The proposed loss function adaptively adjusts the positive sample weight according to the proportion of changed pixels in each batch, enabling the model to sufficiently focus on changed pixels even when changed-region samples are sparse. Meanwhile, multibranch supervision is conducted by combining the main prediction and auxiliary prediction results, which helps reduce the influence of background regions, improve localization accuracy, and further enhance the precision of change detection results.

### 3.2. SAM3-Based Bitemporal Image Encoding with Channel Adapters

This study employs a pre-trained SAM3 visual encoder [17] with frozen parameters to extract multi-level spatial features separately from the two images, thus converting the input images into multi-scale feature representations. Given the bitemporal remote sensing images *T1* and *T2*, denoted as $I_{1}$ and $I_{2}$, respectively, the feature encoding process for either temporal image is formulated as shown in Equation (1) [17]:

(1) $${\left\{F_{s}^{\left(t\right)}\right\}}_{s=1}^{3}=\Phi_{SAM3}(I_{t}),t\in \{1,2\}$$

where $\Phi_{SAM3}$ denotes the SAM3 visual encoder, and $F_{s}^{\left(t\right)}$ represents the spatial feature of the *t*-th temporal image at the *s*-th scale. The last three feature layers generated by SAM3 are selected as multiscale spatial representations, corresponding to 1/8, 1/16, and 1/32 of the input image resolution, respectively. These three feature levels are selected to balance semantic discrimination, spatial detail, and computational cost. The first feature level has a higher spatial resolution and mainly contains low-level visual information, such as textures and edges. It may be more sensitive to illumination differences, local texture variations, and registration noise. Its use would also increase the computational cost of subsequent feature interaction. In contrast, the last three feature levels provide progressively stronger object-structure and semantic representations at different spatial scales. In addition, the decoder directly receives the shallow input formed by concatenating the original bitemporal images, which supplements fine spatial information. Therefore, the three feature levels at 1/8, 1/16, and 1/32 resolutions are used for subsequent change modeling.

Since features at different scales need to be consistent with the channel dimensions required by the subsequent spatial interaction modules, a 1 × 1 channel adaptation layer is introduced. This operation aligns the channel dimensions of the SAM3 features with the subsequent interaction modules while preserving their spatial resolution. The channel adaptation process is formulated as shown in Equation (2):

(2) $$X_{s}^{\left(t\right)}=W_{s}*F_{s}^{\left(t\right)}+b_{s},t\in \{1,2\},s=1,2,3$$

where ∗ denotes the convolution operation, and $W_{s}$ and $b_{s}$ represent the convolution kernel and bias parameter at the *s*-th scale, respectively. After channel adaptation, the numbers of feature channels at the three scales are adjusted to 64, 128, and 256, respectively, resulting in the bitemporal multiscale spatial features $X_{s}^{\left(1\right)}$ and $X_{s}^{\left(2\right)}$. To model the change relationships between bitemporal images, a cross-temporal attention [42] operation is further introduced at each scale, and the resulting spatial change feature is formulated as shown in Equation (3):

(3) $$A_{s}={\mathrm{CrossAtt}}_{s}(X_{s}^{\left(1\right)},X_{s}^{\left(2\right)}),S=1,2,3$$

where denotes the cross-temporal attention operator at the *s*-th scale, and represents the spati ${\mathrm{CrossAtt}}_{s}$ al change feature after bitemporal feature interaction.

Specifically, at the *s*-th scale, $X_{s}^{\left(1\right)}$ and $X_{s}^{\left(2\right)}$ denote the bitemporal features obtained after SAM3 encoding and channel adaptation. A 3 × 3 depthwise convolution is first used for positional encoding. The attention inputs are then obtained through a residual connection and layer normalization, as formulated in Equation (4) [42]:

(4) $${\tilde{X}}_{s}^{\left(t\right)}=LN(X_{s}^{\left(t\right)}+DWConv_{3\times 3}(X_{s}^{\left(t\right)})),t\in \{1,2\}$$

The two temporal features are then divided into $n_{s}\times n_{s}$ non-overlapping windows. A shared linear projection is applied to both temporal features to generate the query, key, and value representations, as formulated in Equation (5) [42]:

(5) $$\left[Q_{s,CT}^{\left(t\right)},K_{s,CT}^{\left(t\right)},V_{s,CT}^{\left(t\right)}]=Split(Win_{n_{s}}({\tilde{X}}_{s}^{\left(t\right)})W_{s,CT}^{QKV}+b_{s,CT}^{QKV}),t\in \{1,2\right\}$$

Here, $Win_{n_{s}}(\cdot )$ denotes the window partition operation, while $W_{s,CT}^{QKV}$ and $b_{s,CT}^{QKV}$ denote the parameters of the shared linear projection.

Average pooling is applied to the query and key features within each window to obtain the window-level representations ${\bar{Q}}_{s,CT}^{\left(t\right)}$ and ${\bar{K}}_{s,CT}^{\left(t\right)}$. For the two interaction directions (a,b)∈{(1,2),(2,1)}, the window correlation is calculated as shown in Equation (6) [42]:

(6) $$R_{s}^{\left(a,b\right)}=\frac{{\bar{Q}}_{s,CT}^{\left(a\right)}{\left({\bar{K}}_{s,CT}^{\left(b\right)}\right)}^{T}}{\sqrt{D_{s}}},(a,b)\in \{(1,2),(2,1)\}$$

Here, a denotes the temporal index of the query feature, b denotes the temporal index of the key and value features, and $D_{s}$ denotes the query and key embedding dimension at the *s*-th scale. For each query window, the $k_{s}$ cross-temporal windows with the highest correlations are selected as shown in Equation (7) [42]:

(7) $$I_{s}^{\left(a,b\right)}=TopKIndex(R_{s}^{\left(a,b\right)},k_{s}),(a,b)\in \{(1,2),(2,1)\}$$

Here, $I_{s}^{\left(a,b\right)}$ denotes the window routing indices. The values of $k_{s}$ at the three scales are set to 8, 16, and 4, respectively. According to the routing indices, the key and value features are gathered from the selected windows of temporal feature *b*. Before gathering, the key and value features in each window are compressed into 4 × 4 tokens through adaptive average pooling, as formulated in Equation (8) [42]:

(8) $$K_{s,g}^{\left(a,b\right)}=Gather(AAP_{4\times 4}(K_{s,CT}^{\left(b\right)}),I_{s}^{\left(a,b\right)}),V_{s,g}^{\left(a,b\right)}=Gather(AAP_{4\times 4}(V_{s,CT}^{\left(b\right)}),I_{s}^{\left(a,b\right)})$$

Fine-grained attention is then calculated between each query window and the selected cross-temporal windows in the multi-head dimension. The attention weights and output features are expressed as shown in Equation (9) [42]:

(9) $$\Omega_{s}^{\left(a,b\right)}=Soft\max(\frac{Q_{s,CT}^{\left(a\right)}{\left(K_{s,g}^{\left(a,b\right)}\right)}^{T}}{\sqrt{D_{S}}}),Y_{s}^{\left(a,b\right)}=\Omega_{s}^{\left(a,b\right)}V_{s,g}^{\left(a,b\right)},(a,b)\in \{(1,2),(2,1)\}$$

Here, $\Omega_{s}^{\left(a,b\right)}$ denotes the cross-temporal attention matrix used to weight the value features. The output $Y_{s}^{\left(2,1\right)}$ is used to update the first temporal branch, while $Y_{s}^{\left(1,2\right)}$ is used to update the second temporal branch. The outputs in both directions are fused with the local positional encoding and residual features of the corresponding branches, followed by output projection and a feed-forward network. Finally, the enhanced bitemporal features are concatenated along the channel dimension and processed by a 3 × 3 convolution, layer normalization, and ReLU activation to obtain the spatial change feature $A_{s}$.

### 3.3. GCSA-Based Spatiotemporal Fused Feature Enhancement for Changed Regions

**Figure 2:**
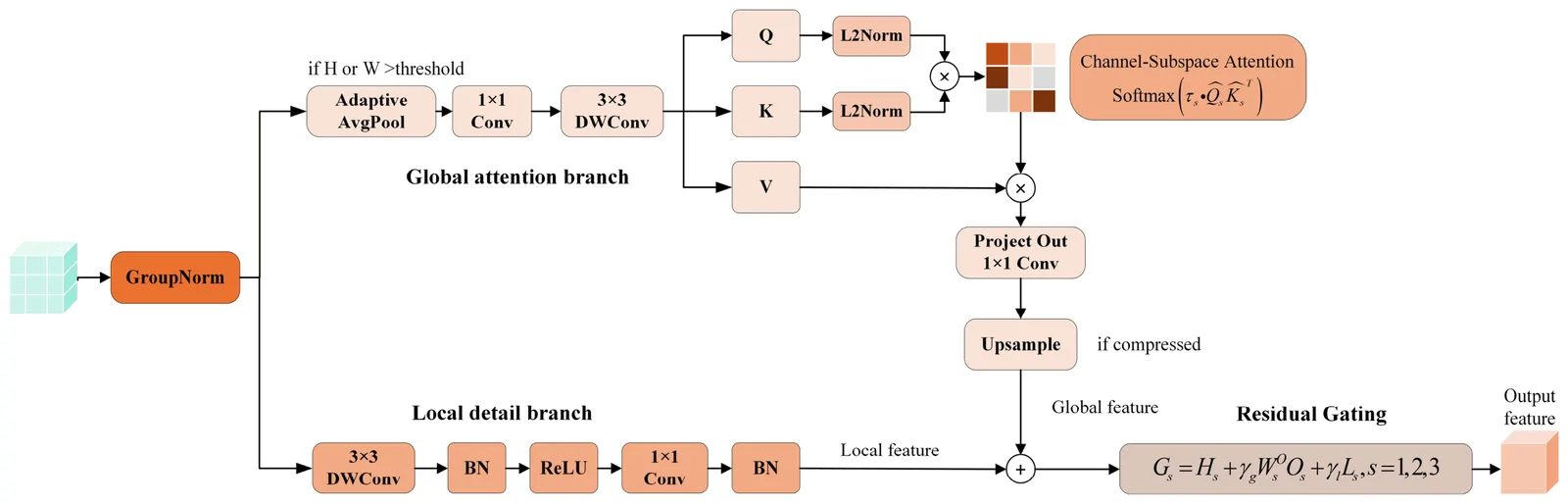
Structure of the global context spatial attention (GCSA) module. GroupNorm, group normalization; AvgPool, average pooling; Conv, convolution; DWConv, depthwise convolution; BN, batch normalization; ReLU, rectified linear unit; L2Norm, L2 normalization; Q, K, and V, query, key, and value features, respectively; H and W, the height and width of the input feature map, respectively.

To effectively fuse spatial change features and temporal evolution features, this paper introduces the GCSA mechanism [18] to further enhance the model’s attention to changed regions, and its specific structure is shown in Figure 2. The multiscale spatial change $A_{s}$ features obtained by cross-temporal attention are aligned with the temporal features $T_{s}$ extracted by the temporal branch in terms of scale and concatenated along the channel dimension, followed by convolution for feature compression and fusion. For the first two scales, the fusion process is formulated in Equation (10):

(10) $$H_{s}=\mathrm{ReLU}(\mathrm{BN}({\mathrm{Conv}}_{3\times 3}^{\left(s\right)}(\mathrm{Concat}(A_{s},T_{s})))),\;s=1,2$$

where $\mathrm{Concat}\left(\cdot \right)$ denotes concatenation along the channel dimension, ${\mathrm{Conv}}_{3\times 3}^{\left(s\right)}(\cdot )$ represents the 3 × 3 convolution mapping at the *s*-th scale, BN(⋅) denotes the normalization operation, and ReLU(⋅) represents the nonlinear activation function. For the highest semantic scale, only the spatial feature is used to obtain the multiscale spatiotemporal fused feature, as formulated in Equation (11):

(11) $$H_{3}=\mathrm{ReLU}(\mathrm{BN}({\mathrm{Conv}}_{3\times 3}^{\left(3\right)}(A_{3})))$$

The spatiotemporally fused feature $H_{s}$ jointly encodes cross-temporal spatial change responses and temporal evolution information. GCSA performs global dependency modeling and local detail enhancement on this joint representation to improve response consistency within changed regions and boundary representation. First, the input feature $H_{s}\in {\mathbb{R}}^{C_{s}\times H_{s}\times W_{s}}$ at the *s*-th scale is normalized by GroupNorm. A shared 1 × 1 convolution is then used to jointly generate the query, key, and value features, which are formulated in Equation (12) [18,43]:

(12) $$H_{s}^{N}=GN(H_{s}),Q_{s}=W_{s}^{Q}*H_{s}^{N},K_{s}=W_{s}^{K}*H_{s}^{N},V_{s}=W_{s}^{V}*H_{s}^{N},\;s=1,2,3$$

where $H_{s}^{N}$ denotes the normalized fused feature, and $W_{s}^{Q},W_{s}^{K},W_{s}^{V}$ are the convolution parameters at the *s*-th scale. Then, a 3 × 3 depthwise convolution is used to introduce local spatial context, producing locally enhanced query, key, and value features, which are formulated in Equation (13) [18,43]:

(13) $$\tilde{Q_{s}}={\mathrm{DWConv}}_{3\times 3}(Q_{s}),\;\tilde{K_{s}}={\mathrm{DWConv}}_{3\times 3}(K_{s}),\tilde{V_{s}}={\mathrm{DWConv}}_{3\times 3}(V_{s})$$

To improve the stability of attention computation, $\tilde{Q_{s}}$ and $\tilde{K_{s}}$ are *L2*-normalized, and a learnable temperature parameter $\tau_{s}$ is used to regulate the attention distribution. Global attention matrix at the *s*-th scale can be expressed in Equation (14) [18,43]:

(14) $$M_{S}=\mathrm{Softmax}\left(\tau_{s}\cdot \hat{Q_{s}}{\hat{K_{s}}}^{T}\right)$$

where $\hat{Q_{s}}$ and $\hat{K_{s}}$ denote the normalized query and key features, respectively. The resulting attention matrix models correlations among channel subspaces based on globally aggregated spatial responses rather than explicitly constructing a pixel-wise spatial attention map, while spatial details are further encoded by the preceding 3 × 3 depthwise convolution and the local enhancement branch. Based on the attention matrix, the value feature is weighted and aggregated in Equation (15):

(15) $$O_{s}=M_{s}\tilde{V_{s}}$$

Subsequently, local enhancement is performed in Equation (16):

(16) $$L_{s}=\mathrm{Local}\left(H_{s}\right)$$

where $L_{s}$ denotes the local detail feature extracted by the local enhancement branch, which is used to preserve the boundaries of changed regions and the details of small targets. Finally, the GCSA output feature is obtained through channel output projection and residual connection, which is formulated in Equation (17):

(17) $$G_{s}=H_{s}+\gamma_{g}W_{s}^{O}O_{s}+\gamma_{l}L_{s},\;s=1,2,3$$

where $W_{s}^{O}$ denotes the output projection parameter; $\gamma_{g}$ and $\gamma_{l}$ represent the residual weights of the global attention branch and the local enhancement branch, respectively; and $G_{s}$ denotes the multiscale enhanced feature after global dependency modeling, local detail enhancement, and feature recalibration. Finally, the multiscale features enhanced by GCSA and the shallow input feature obtained by concatenating the original bitemporal images $I_{1}$ and $I_{2}$ are jointly fed into the hierarchical decoder. $I_{1}$ and $I_{2}$ are first concatenated along the channel dimension to obtain the shallow input feature $F_{0}$, after which the decoder progressively fuses features from high semantic levels to low semantic levels. Meanwhile, the high-level temporal feature $F_{v}$ from the temporal branch is processed by 1 × 1 convolution, upsampling, and Sigmoid activation to obtain the auxiliary prediction result $P_{v}$ of the temporal branch, which is formulated in Equation (18) [41]:

(18) $$F_{0}=\mathrm{Concat}(I_{1},I_{2}),P_{v}=\sigma (\mathrm{Up}({\mathrm{Conv}}_{1\times 1}(F_{v})))$$

Finally, the final change probability map P and the intermediate auxiliary prediction results are obtained in Equation (19):

(19) $$\left(P,P_{d}^{1},P_{d}^{2},P_{d}^{3})=\mathrm{Decoder}(G_{1},G_{2},G_{3},F_{0}\right)$$

where $G_{1}$, $G_{2}$, $G_{3}$ denote the scale features enhanced by GCSA, respectively. The decoder performs progressive fusion starting from the largest semantic scale to obtain the final change probability map P, where $P_{d}^{1}$, $P_{d}^{2}$, $P_{d}^{3}$ represent the auxiliary prediction results produced by the intermediate decoder layers.

### 3.4. Dynamic Weighted Loss Function

Considering that in practical change detection tasks, changed regions are usually far fewer than unchanged regions, the model tends to be biased toward background-class prediction during training. Therefore, a dynamic weighted loss function is proposed for both the final prediction map and the multiscale auxiliary prediction results to alleviate this problem. Assume that the pixel set of the *n*-th batch is $\Omega_{n}$, and the changed-pixel set is $\Omega_{n}^{+}$. The proportion of changed pixels in the current batch is denoted as $r_{n}=\frac{\left|\Omega_{n}^{+}\right|}{\left|\Omega_{n}\right|}$, and the dynamic positive sample weight is defined in Equation (20):

(20) $$\lambda_{n}=min(\lambda_{max},max(\lambda_{min},\frac{1}{r_{n}+\epsilon })),\lambda_{max}=15,\lambda_{\min}=5$$

where ϵ is a stability term and is set to 10^−8^. The weight is recalculated for each mini-batch according to the current proportion of changed pixels and constrained to the range [5, 15]. Based on this weight, the dynamic weighted BCE loss is expressed in Equation (21):

(21) $$L_{WBCE}=-\frac{1}{\left|\Omega_{n}\right|}\sum_{i\in \Omega_{n}}\left[\lambda_{n}y_{i}\log p_{i}+(1-y_{i})\log(1-p_{i})\right]$$

The Dice loss [20] is defined in Equation (22):

(22) $$L_{Dice}=1-\frac{2\sum_{i\in \Omega_{n}}p_{i}y_{i}+\epsilon }{\sum_{i\in \Omega_{n}}p_{i}+\sum_{i\in \Omega_{n}}y_{i}+\epsilon }$$

The supervised prediction set, the loss corresponding to each prediction, and the total loss are defined in Equation (23):

(23) $$P=\{P,P_{v},P_{d}^{1},P_{d}^{2},P_{d}^{3}\},L_{m}=L_{WBCE}(P^{m},Y)+L_{Dice}(P^{m},Y),L_{total}=\sum_{m=1}^{5}\beta_{m}L_{m}$$

where $P^{m}\in P$, and $\beta_{m}$ represents the loss weight corresponding to each supervised output. The main prediction is assigned the highest weight, $\beta_{1}=1.0$. To reduce the excessive influence of coarse-scale auxiliary outputs while preserving the dominant role of the final prediction, progressively decreasing weights are adopted and empirically set to 0.6, 0.4, 0.3, and 0.2, respectively. The proposed loss can dynamically adjust the supervision strength of the changed class according to the proportion of changed pixels within each batch. It increases the positive sample loss weight when changed regions are sparse and avoids excessive weight amplification when the proportion of changed regions is relatively high, thereby alleviating the background-class bias caused by class imbalance and improving the localization accuracy and segmentation completeness of changed regions.

## 4. Results and Discussion

### 4.1. Experimental Datasets and Evaluation Metrics

To validate the effectiveness of the method proposed in this paper for change detection in remote sensing images, we conducted experiments using two publicly available change detection datasets: the Wuhan University Change Detection (WHU-CD) dataset [44] and the Sun Yat-sen University Change Detection (SYSU-CD) dataset [45]. These two datasets differ in terms of image spatial resolution, types of change areas, and scene complexity, allowing for a comprehensive evaluation of the model’s adaptability in scenarios involving building change detection and complex feature change detection. The experiments analyzed the model’s performance in three aspects: quantitative metrics, visualization results, and ablation studies.

#### 4.1.1. Experimental Datasets

(1) WHU-CD Dataset. The WHU-CD dataset was released by Ji et al. [44] as part of their research on fully convolutional networks for building extraction from open aerial and satellite imagery. It consists of two large-scale aerial remote sensing images acquired in Christchurch, New Zealand, in 2012 and 2016, respectively, corresponding to a four-year interval. Each image measures 32,507 × 15,354 pixels with a spatial resolution of 0.2 m and records building changes during the post-earthquake reconstruction period. This dataset is primarily intended for building change detection tasks. While the boundaries of the areas of change are relatively clear, there are significant differences in building scale, placing high demands on the model’s ability to represent features at multiple scales and to accurately locate boundaries. The bitemporal images and their corresponding labels were cropped into non-overlapping 256 × 256 image pairs and divided into training, validation, and test sets at a ratio of 8:1:1, containing 6096, 762, and 762 samples, respectively.
(2) SYSU-CD Dataset. The SYSU-CD dataset was introduced by Shi et al. [45] in their research on a deeply supervised attention metric-based network for remote sensing change detection. It contains 20,000 pairs of orthorectified aerial remote sensing images acquired in Hong Kong between 2007 and 2014, each measuring 256 × 256 pixels with a spatial resolution of 0.5 m, covering a variety of typical land cover types such as buildings, roads, and vegetation. Compared to the WHU-CD dataset, the SYSU-CD dataset features a greater variety of change types and more complex scene backgrounds. Differences in illumination, shading, and land cover texture have a more pronounced impact on change detection results, making it better suited for evaluating the model’s generalization ability in complex scenes. In this study, the 20,000 image pairs were divided into training, validation, and test sets at a ratio of 3:1:1, containing 12,000, 4000, and 4000 samples, respectively. The same fixed data splits were used in all experiments.

#### 4.1.2. Experimental Settings

The proposed model was implemented on Ubuntu (Version 22.04) using Python (Version 3.12), PyTorch (Version 2.8.0), and CUDA (Version 12.8), and was trained on a single NVIDIA GeForce RTX 4090 D GPU with 24 GB of memory. The experimental platform was equipped with a 16-core Intel Xeon Platinum 8481C CPU and 80 GB of RAM. The frozen SAM3 visual encoder contained 449.01 M parameters, while the remaining 7.03 M parameters of SAM3TGNet were trainable. Each input sample consisted of a pair of bitemporal RGB images ($T_{1}$ and $T_{2}$) and the corresponding binary change label. After conversion to tensors, the bitemporal RGB images were normalized using a mean and standard deviation of [0.5, 0.5, 0.5]. During training, synchronized random horizontal flipping, vertical flipping, scale-based random cropping, and Gaussian blurring were applied to the paired images, with the same geometric transformations applied to the labels. Scale-based random cropping was used to vary the relative sizes and local spatial contexts of changed regions within the fixed-size network input, thereby helping the model learn change patterns at different scales. Gaussian blurring was used to introduce moderate variations in image sharpness and reduce excessive reliance on high-frequency texture cues, thereby improving robustness to image-quality variations in bitemporal remote sensing observations. No random data augmentation was applied to the validation or test samples. No random data augmentation was applied to the validation or test samples. The batch size and number of training epochs were set to 16 and 200, respectively. The model was optimized using SGD with an initial learning rate of 0.003 and a linear learning-rate decay strategy. The number of data-loading workers was set to 2, and the number of change detection classes was set to 2.

#### 4.1.3. Evaluation Metrics

To quantitatively evaluate the change detection performance of the method described in this paper, we use precision (Pre), recall (Rec), F1-score (F1), and intersection over union (IoU) as the primary evaluation metrics. In this study, regions with changes are classified as the positive class, and regions without changes are classified as the negative class. The pixel-level confusion matrix is shown in Table 1.

(1) Precision (Pre): Precision represents the proportion of pixels that actually changed among those predicted to have changed in change detection results. It reflects the model’s ability to control false positives. The formula is in Equation (24) [45]:
  (24) $$Pre=\frac{TP}{TP+FP}$$
(2) Recall (Rec): Recall represents the proportion of correctly detected changing pixels in the change detection prediction results relative to the total number of actually changing pixels. It is calculated in Equation (25) [45]:
  (25) $$Rec=\frac{TP}{TP+FN}$$
(3) F1-score (F1): In a binary change detection task, the changed class is designated as class 1, and the unchanged class is designated as class 0. In this study, the F1 score for the changed class is denoted as F1, and its calculation formula is in Equation (26) [45]:
  (26) $$F1=\frac{2TP}{2TP+FP+FN}$$
(4) Intersection over Union (IoU): IoU is used to measure the degree of overlap between the predicted region and the ground-truth region. Its formula is in Equation (27) [45]:
  (27) $$IoU=\frac{TP}{TP+FN+FP}$$
(5) Boundary F1-score (BF1): BF1 is used to measure the matching accuracy between the predicted boundary and the ground-truth boundary. It jointly considers boundary precision and boundary recall. The formulas are given in Equations (28) and (29) [46]:
  (28) $$P_{B}=\frac{N_{PG}}{N_{P}},R_{B}=\frac{N_{GP}}{N_{G}}$$
  (29) $$BF1=\frac{2P_{B}R_{B}}{P_{B}+R_{B}}$$
  where $N_{P}$ and $N_{G}$ denote the numbers of predicted and ground-truth boundary pixels, respectively; $N_{PG}$ and $N_{GP}$ denote the numbers of matched boundary pixels within the tolerance ε. In this study, ε is set to 4 pixels.
(6) Boundary Intersection over Union (BIoU): BIoU is used to measure the degree of overlap between the predicted boundary region and the ground-truth boundary region. Its formula is in Equation (30) [46]:
  (30) $$BIoU=\frac{\left|\left.B_{d}\left(P\right)\cap B_{d}\left(G\right)\right|\right.}{\left.{\left|B\right.}_{d}\left(P\right)\cup B_{d}\left(G\right)\right|}$$
  where $B_{d}\left(P\right)$ and $B_{d}\left(G\right)$ denote the boundary regions of the predicted result and the ground truth, respectively, and d denotes the boundary width. In this study, the boundary width ratio is set to 0.02.

### 4.2. Experimental Result Analysis

#### 4.2.1. Quantitative Analysis

**Figure 3:**
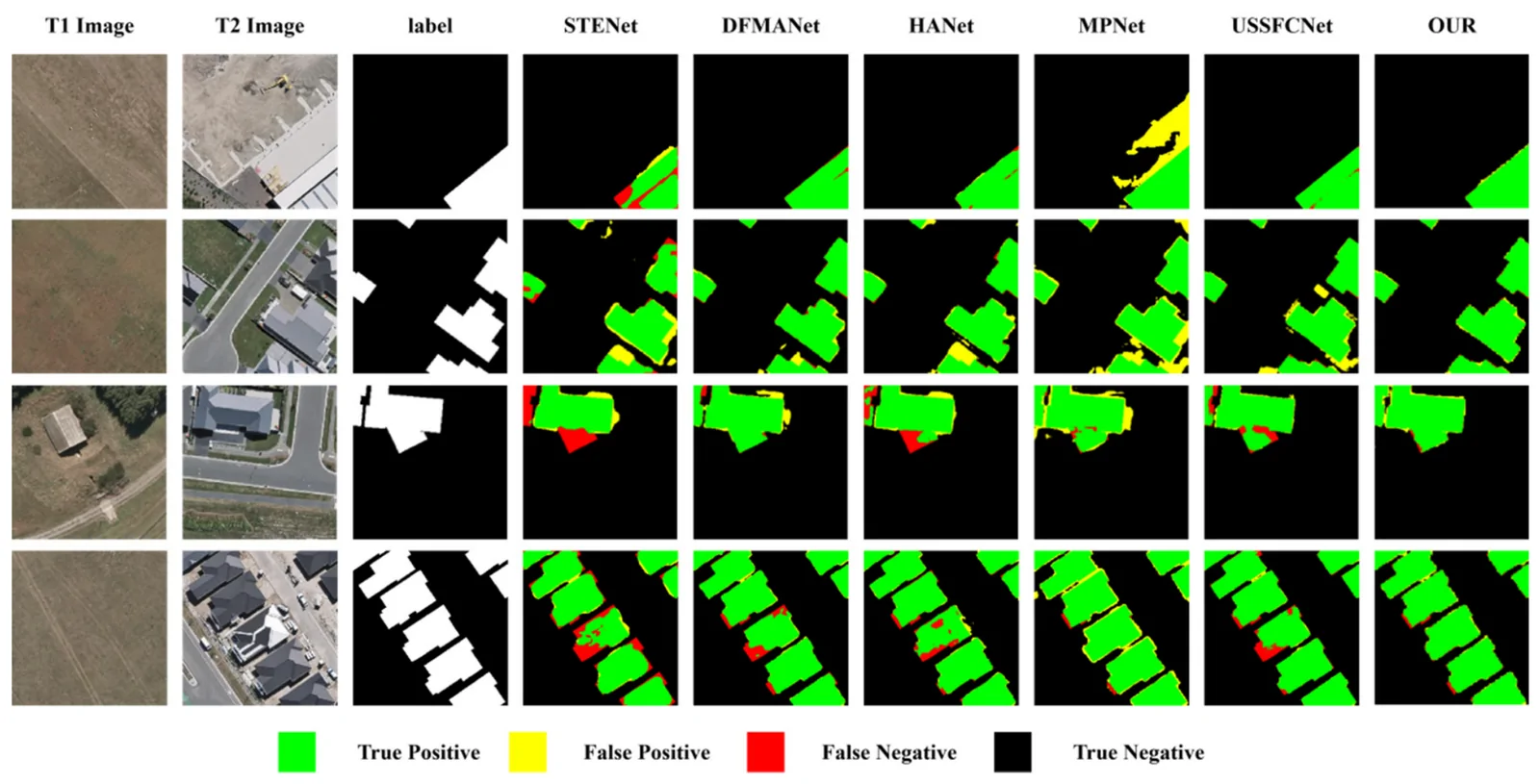
Comparison results of different change detection methods on the WHU-CD dataset.

**Figure 4:**
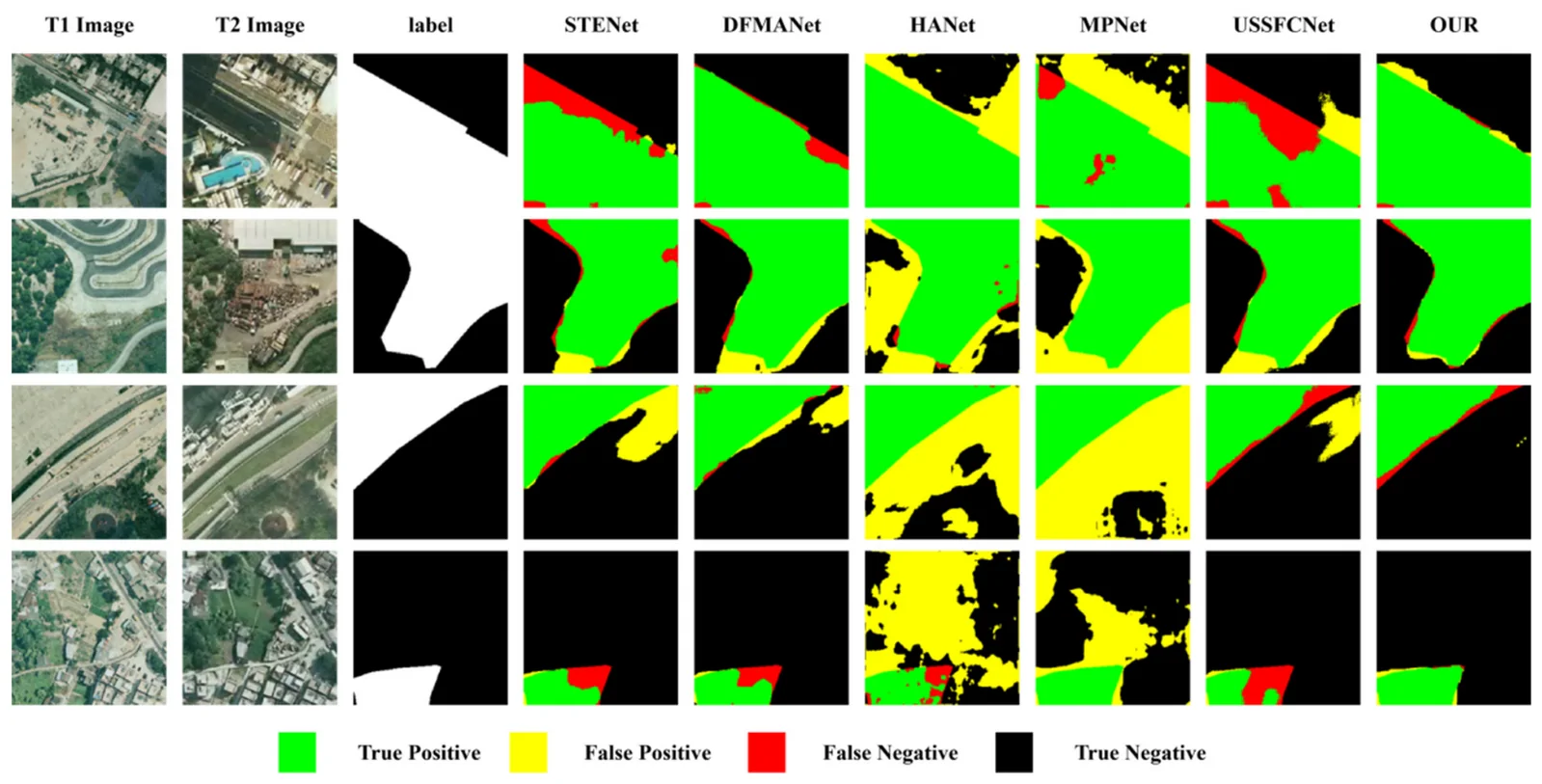
Comparison results of different change detection methods on the SYSU-CD dataset.

To validate the effectiveness of the proposed method, comparative experiments are conducted on the WHU-CD and SYSU-CD datasets with several representative methods. The methods used for comparison cover CNN-based change detection methods, as well as detection methods that integrate both CNN and Transformer. Pre, Rec, F1, and IoU are adopted to evaluate different methods. To reduce the effects of random initialization and training fluctuations on the experimental results, SAM3TGNet was independently trained and tested three times on the WHU-CD and SYSU-CD datasets. The mean and standard deviation of Pre, Rec, F1, and IoU are reported in Table 2 and Table 3. All quantitative evaluation metrics were calculated using the complete test sets, whereas Figure 3 and Figure 4 present only representative samples for a qualitative comparison of the detection results obtained by different methods.

(1) Quantitative analysis on WHU-CD: As shown in Table 2, the proposed method achieves a mean F1-score of 94.08 ± 0.12% and an IoU of 88.82 ± 0.22% on the WHU-CD dataset, outperforming all comparison methods. Compared with the baseline model STENet, our method achieves a 5.43 percentage point increase in F1 score and a 9.22 percentage point increase in IoU. This indicates that our model has achieved significant improvements in both its overall recognition capability in areas with building changes and the degree of spatial overlap. In terms of the Pre and Rec metrics, our method achieved 94.14 ± 0.13% and 94.02 ± 0.15%, respectively, with the two being essentially on par. The small standard deviations indicate stable performance across the three independent runs. This indicates that the model is able to effectively control false negatives while reducing false positives, demonstrating well-balanced change detection capabilities. Compared with other methods, DFMANet achieves an F1 score of 92.60% and an IoU of 86.23% on the WHU-CD dataset. Although it shows some advantages, these metrics remain lower than those of the method proposed in this study. MPNet achieved the Rec of 93.72%, indicating strong detection capability for changed regions. However, its Pre was only 84.93%, suggesting that this method tends to misclassify unchanged regions as changed ones, resulting in a high number of false positives. In contrast, our method significantly improves F1 and IoU while maintaining a high recall rate, indicating that the multiscale semantic features provided by the SAM3 feature encoder enhance the model’s ability to represent building changed regions, and that the GCSA module, after spatiotemporal fusion, further improves the localization of change region boundaries and regional integrity. Consequently, the improvements achieved by our method on the WHU-CD dataset are reflected not only in comprehensive metrics such as F1 and IoU but also in a more balanced detection performance between Pre and Rec.
(2) Quantitative analysis on SYSU-CD: As shown in Table 3, the proposed method achieved a mean F1 score of 82.91 ± 0.20% and a mean IoU of 70.81 ± 0.29%, both of which were the highest among all compared methods. Compared with STENet, the proposed method improved the F1 score by 5.26 percentage points and the IoU by 7.35 percentage points, indicating that it can still achieve stable performance gains in complex and dynamic scenarios. The SYSU-CD dataset contains a wide variety of variation types and features highly complex scenes and interference, placing higher demands on a model’s generalization ability and its capacity to recognize changes in complex ground features. In terms of the Pre and Rec metrics, DFMANet achieved a Pre of 83.57%, indicating its strong ability to control false positives. MPNet achieves a recall of 82.97%, demonstrating its strong sensitivity to changed regions. However, DFMANet’s Rec and MPNet’s Pre are lower than those of the method proposed in this study, indicating that both methods still exhibit a certain imbalance between false positive and false negative control. In contrast, our method achieves mean Pre and Rec values of 82.75 ± 0.23% and 83.06 ± 0.17%, respectively. Although its Pre is not the highest, its Rec is the highest among all methods, and its mean F1 and IoU reach 82.91 ± 0.20% and 70.81 ± 0.29%, respectively—both of which are the highest mean values. This indicates that this method is able to effectively control false positives while ensuring the detection capability for regions of change, thereby delivering more stable performance in terms of overall change detection accuracy and region overlap. The proposed method does not merely improve the detection rate of changed pixels, but rather achieves more stable overall performance in terms of false positive control, false negative control, and area overlap. This demonstrates that the method can more accurately localize the spatial extent of changed areas in complex land cover change scenarios. A comprehensive analysis of the quantitative results from the two datasets reveals that SAM3 feature encoding, spatiotemporal feature fusion, and GCSA attention enhancement exhibit good synergy, delivering stable performance gains in both building change scenarios and complex land cover change scenarios.

#### 4.2.2. Qualitative Analysis

To further analyze the differences among various methods in terms of change region localization and boundary preservation, this study visualizes the detection results using a pixel-level color-coding scheme. True positives (TP) are represented in green, false positives (FP) in yellow, true negatives (TN) in black, and false negatives (FN) in red. A more complete green area indicates more thorough coverage of the actual changed regions, a smaller yellow area indicates fewer false positives, and a smaller red area indicates fewer false negatives. Therefore, the visualizations clearly illustrate the differences among the various methods in terms of the integrity of the regions of change, boundary localization, and suppression of spurious changes. Figure 3 and Figure 4 show the visual comparison results of different methods on the two datasets, respectively.

(1) Visualization analysis on WHU-CD: As shown in Figure 3, the comparison results on the WHU-CD dataset indicate that USSFCNet and HANet produce more red regions along building edges and corners, showing discontinuities or fragmented results. This indicates that these two methods are insufficient at detecting change boundaries and tend to fragment areas of change. DFMANet and MPNet produce more complete green regions, but yellow false-positive regions appear in unchanged areas such as shadows and open spaces, mistaking changes in lighting for actual changes in buildings. In comparison, the proposed method achieves the best overall continuity and integrity of the green areas, and preserves building outlines more effectively than other methods. At the same time, false positives in the yellow areas are significantly reduced, indicating that the proposed method further enhances the understanding of spatial context, better distinguishes between genuine changes and noise, and is effective in both preserving building boundaries and suppressing spurious changes.
(2) Visualization analysis on SYSU-CD: As shown in Figure 4, the green regions produced by USSFCNet and HANet are relatively scattered and have more red regions, indicating that these two methods do not sufficiently cover changed regions. The results of HANet and MPNet contain a large number of yellow false positives, where changes in lighting or shadows are mistakenly identified as changes. In contrast, the green regions identified by the method proposed in this paper are more continuous, and both the yellow false positives and red false negatives are significantly fewer than those of the other comparison methods. This shows that the proposed method is effective at suppressing spurious variations and is better suited for regions with multiscale variations. Overall, the visualization results are consistent with the quantitative metrics, validating the effectiveness of our method in change detection.

#### 4.2.3. Failure-Case Analysis

**Figure 5:**
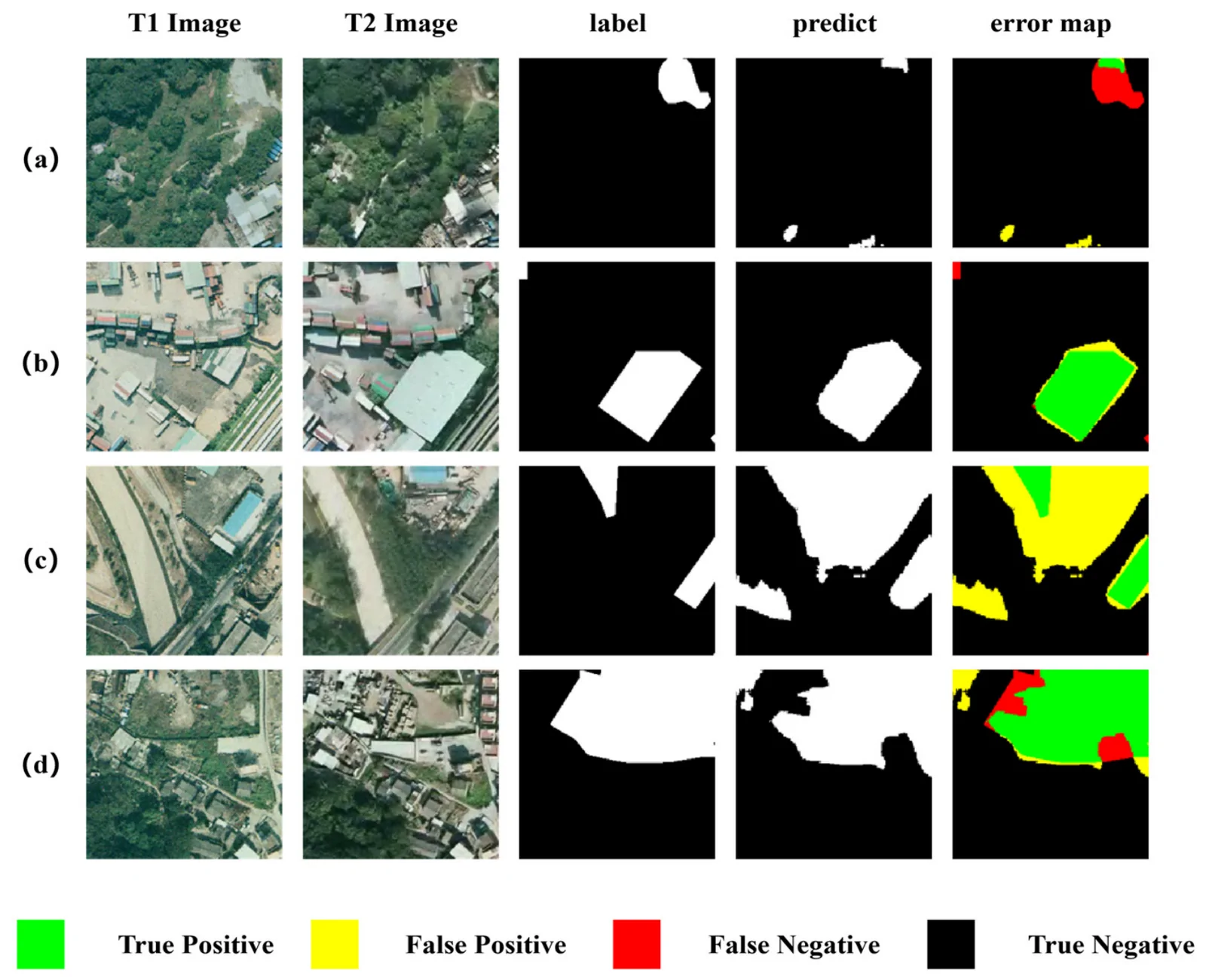
Representative failure cases of SAM3TGNet under challenging conditions. From top to bottom: (**a**) illumination and shadow interference; (**b**) missed detection of a very small changed object; (**c**) vegetation-induced pseudo-change interference; (**d**) boundary localization errors in a densely built-up area. From left to right are the T1 image, T2 image, ground truth, prediction, and error map. In the error maps, green, yellow, red, and black denote true positives, false positives, false negatives, and true negatives, respectively.

As shown in the previous visualization results, SAM3TGNet can extract change regions relatively completely in most scenes. It can also effectively reduce pseudo-change interference caused by shadows, illumination differences, and complex backgrounds, while maintaining good regional continuity and boundary localization. However, some local residual errors still occur in a few more challenging scenes, as shown in Figure 5. In Figure 5a, when shadows, vegetation, and multiple types of ground-object interference occur simultaneously, some local appearance differences may produce feature responses similar to those of real changes, resulting in a small number of false detections or missed detections. In Figure 5b, the responses of very small changed objects may be weakened during multiscale feature extraction. Their detailed information may therefore be difficult to fully recover during subsequent feature fusion and decoding. In Figure 5c, big temporal differences in vegetation produce a small number of pseudo-change responses in local regions. In Figure 5d, the large building change region generally maintains good integrity, but a small number of boundary deviations still occur in local areas with densely distributed buildings and closely adjacent boundaries. Overall, these errors mainly occur under extreme conditions with multiple interfering factors, very small target scales, or highly complex local structures. The main changed regions can still be detected accurately and continuously. This demonstrates the good robustness of the proposed method in complex scenes and also provides a direction for further improving fine-grained feature representation under extreme conditions.

#### 4.2.4. Computational Complexity and Inference Efficiency Analysis

To evaluate the computational efficiency of different methods, all models were tested on an NVIDIA GeForce RTX 4090D with input consisting of two 256 × 256 bi-temporal images, a batch size of 1, and using FP32 precision. FPS was calculated over 300 repeated forward passes after 50 warm-up iterations, excluding data loading time; FLOPs were computed using THOP [49] according to its MACs-based statistical standards. As shown in Table 4, USSFCNet has the fewest trainable parameters and the lowest FLOPs, while STENet has the highest inference speed. SAM3TGNet only has 7.03 M trainable parameters, but due to the frozen SAM3 visual encoder, its frozen parameter count and computation are relatively large, resulting in an FPS of 32.49. Overall, SAM3TGNet gains stronger pretrained feature representation by adding some computational overhead, but there is still room to optimize its model size and inference efficiency.

### 4.3. Ablation Studies

To further validate the impact of each of the proposed improvements on model performance, this paper uses the STENet method as a baseline and progressively incorporates the dynamic weight loss, SAM3 feature encoder, and GCSA modules, conducting ablation experiments on the WHU-CD and SYSU-CD datasets. The results are shown in Table 5. All ablation experiments were conducted under exactly the same experimental settings, and only the module under evaluation was changed. Table 5 reports the results of a single controlled run under the same experimental settings.

**Figure 6:**
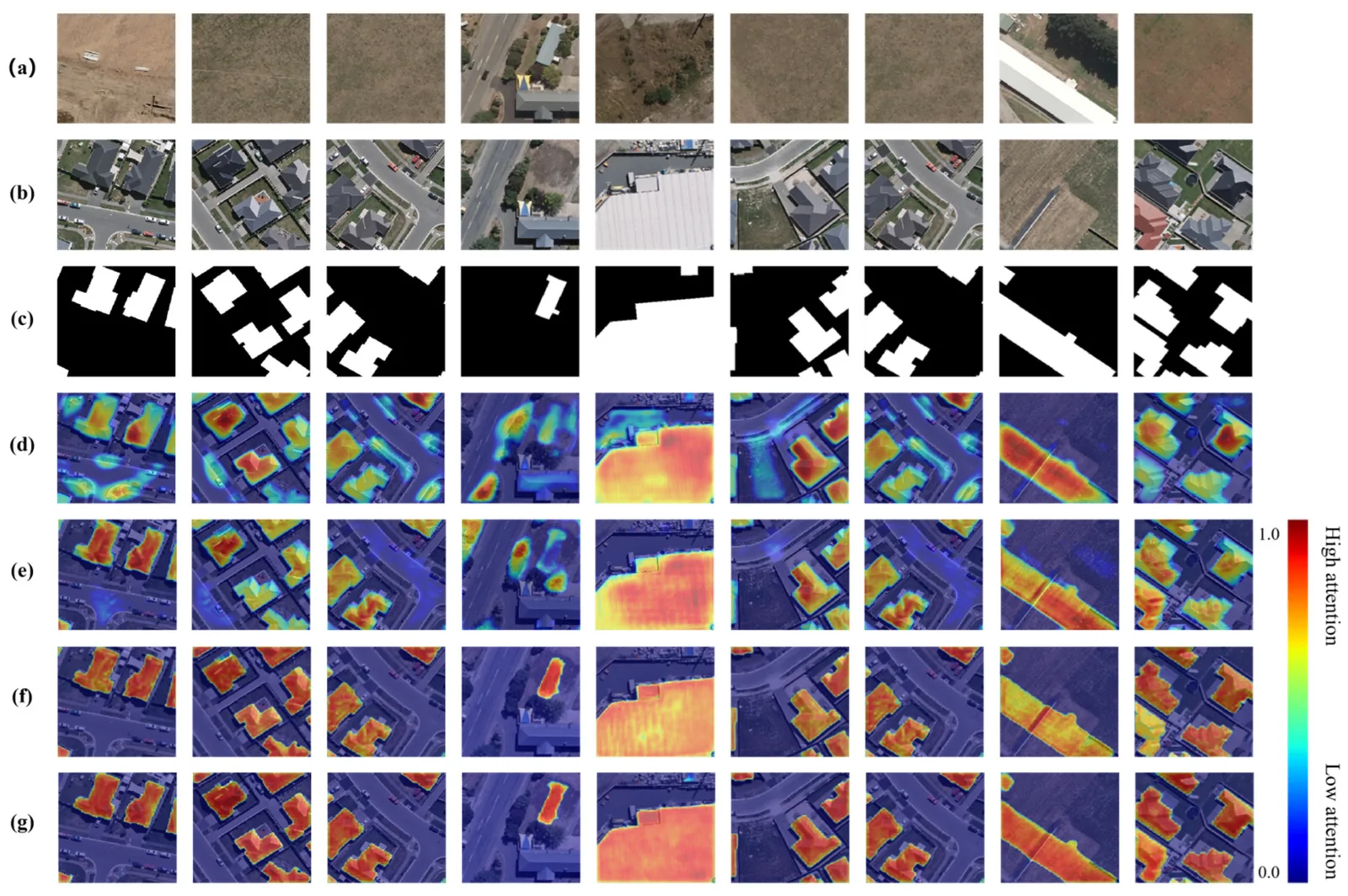
Visualization examples of heatmaps for each ablation variant in the WHU-CD dataset. The rows from top to bottom are: (**a**) T1 image. (**b**) T2 image. (**c**) Ground truth. (**d**) Variant1. (**e**) Variant2. (**f**) Variant3. (**g**) The proposed SAM3TGNet. The color bar represents the normalized Grad-CAM response intensity, ranging from 0 to 1 without a physical unit; red indicates a higher relative response, whereas blue indicates a lower relative response.

**Figure 7:**
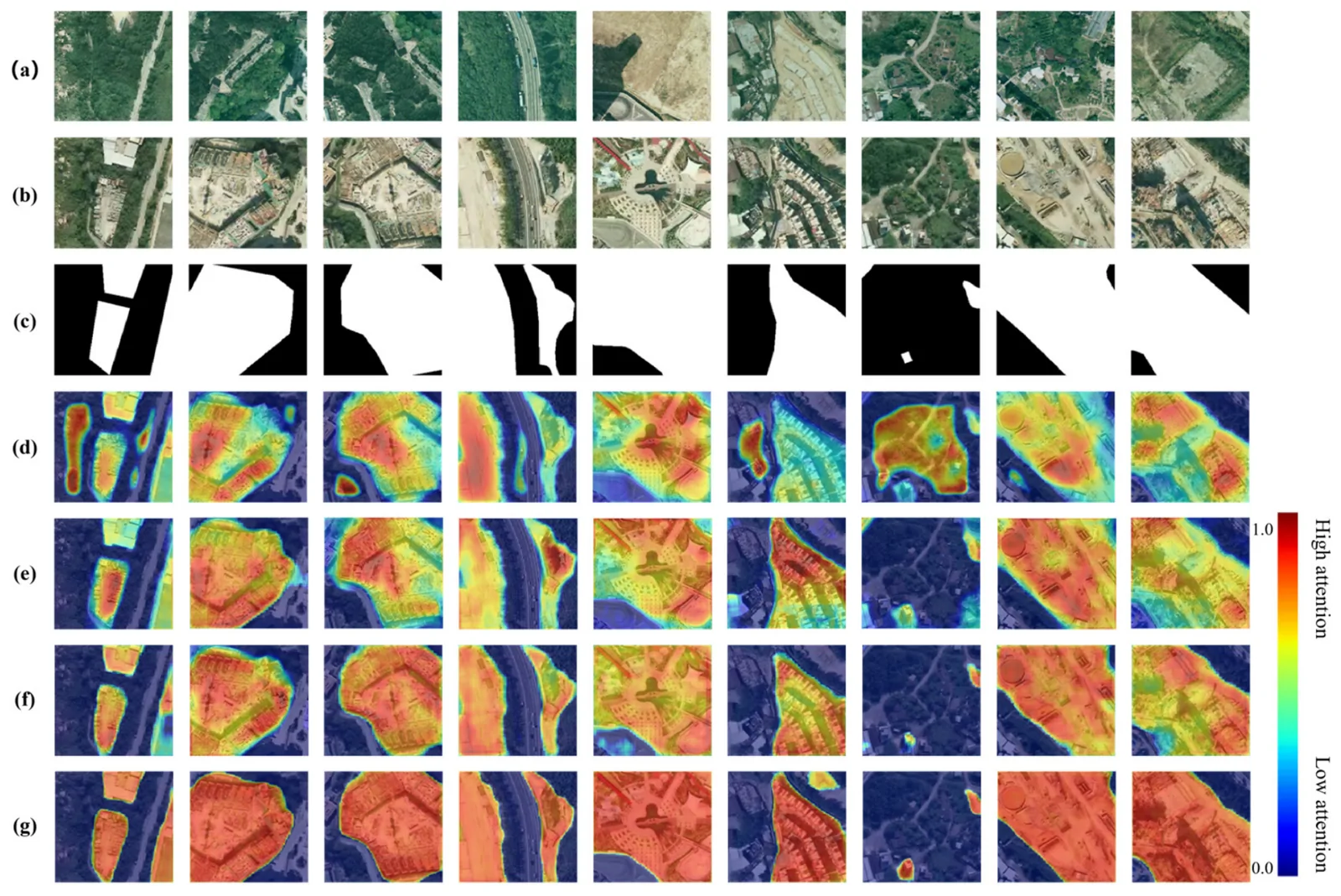
Visualization examples of heatmaps for each ablation variant in the SYSU-CD dataset. The rows from top to bottom are: (**a**) T1 image. (**b**) T2 image. (**c**) Ground truth. (**d**) Variant1. (**e**) Variant2. (**f**) Variant3. (**g**) The proposed SAM3TGNet. The color bar represents the normalized Grad-CAM response intensity, ranging from 0 to 1 without a physical unit; red indicates a higher relative response, whereas blue indicates a lower relative response.

(1) Effect of the dynamic weighted loss: As shown in Table 5 and Table 6, after incorporating dynamic weight loss into the original STENet, the F1 score on the WHU-CD dataset increased from 88.65% to 91.73%, and the IoU increased from 79.60% to 84.72%, representing increases of 3.08 percentage points and 5.12 percentage points, respectively; On the SYSU-CD dataset, the F1 score increased from 77.65% to 78.45%, and the IoU increased from 63.46% to 64.54%, representing increases of 0.80 percentage points and 1.08 percentage points, respectively. Furthermore, as shown in Table 6, standard BCE + Dice achieves F1 scores of 90.56% and 78.04% and IoU scores of 82.75% and 63.99% on the WHU-CD and SYSU-CD datasets, respectively. Compared with standard BCE + Dice, the proposed dynamically weighted BCE + Dice improves F1 by 1.17 and 0.41 percentage points and IoU by 1.97 and 0.55 percentage points on the two datasets, respectively. These results indicate that, beyond the joint supervision of BCE and Dice, dynamically adjusting the positive-class weight according to the proportion of changed pixels in each batch can further mitigate the effect of class imbalance during training. In contrast, the improvement achieved by dynamic weight loss on the WHU-CD dataset is more pronounced. This may be because the WHU-CD dataset is primarily focused on building change detection, where the types of changing objects are relatively concentrated, and the impact of class imbalance on model training is more direct. However, the SYSU-CD dataset contains a wide variety of land cover change types, with more complex scene backgrounds and interfering factors. Relying solely on loss weight adjustments is insufficient to adequately address the challenges of representing semantic differences and distinguishing complex backgrounds. Therefore, the improvement achieved by adding a dynamic weight loss term to the SYSU-CD dataset alone is relatively limited.
(2) Effect of the SAM3 feature encoder: After further incorporating the SAM3 feature encoder on top of the dynamic weighted loss, the F1 score and IoU on the WHU-CD dataset increased to 92.95% and 86.82%, respectively, representing improvements of 1.22 percentage points and 2.10 percentage points over the previous stage. On the SYSU-CD dataset, the F1 score and IoU increased to 80.78% and 67.75%, respectively, representing improvements of 2.33 percentage points and 3.21 percentage points over the previous stage. Compared with the dynamic weighted loss, the SAM3 feature encoder achieves more significant improvements on the SYSU-CD dataset, indicating that its multiscale semantic representation capability can better adapt to scenes with complex changes.
(3) Effect of the GCSA module: After further introducing the GCSA module on top of the SAM3 feature encoder and dynamically weighted loss, the F1 score on the WHU-CD dataset increased from 92.95% to 94.16%, and the IoU increased from 86.82% to 88.96%, representing improvements of 1.21 and 2.14 percentage points, respectively. On the SYSU-CD dataset, the F1 score increased from 80.78% to 82.93%, and the IoU increased from 67.75% to 70.84%, representing improvements of 2.15 and 3.09 percentage points, respectively. These results indicate that the GCSA module can further enhance the model’s ability to distinguish changed from unchanged regions based on the semantic features extracted by the SAM3 encoder. This is because, although the SAM3 feature encoder has already enhanced the multiscale semantic representation of bitemporal images, the fused features may still contain background noise, spurious change responses, and ambiguous boundary information after cross-temporal interaction and spatiotemporal fusion. If these fused features are not further refined, the model is prone to issues such as discontinuous responses within changed regions, inaccurate boundary localization, and false detections in complex backgrounds. When GCSA is applied to the features resulting from spatiotemporal fusion, it enhances the correlations between different spatial locations within the same changed region through global contextual relationships, making the responses within changed regions more continuous and complete. At the same time, it preserves change boundaries and fine-scale structural information through local spatial details, ensuring that the prediction results better align with the actual changed regions. To further quantitatively evaluate this boundary-enhancement effect, Boundary F1-score (BF1) and Boundary Intersection over Union (BIoU) were additionally adopted as boundary-sensitive evaluation metrics, with the results shown in Table 7. After introducing GCSA, BF1 on the WHU-CD dataset increased from 81.68% to 87.51%, while BIoU increased from 65.49% to 71.67%, corresponding to improvements of 5.83 and 6.18 percentage points, respectively. On the SYSU-CD dataset, BF1 increased from 48.47% to 50.54%, and BIoU increased from 35.81% to 37.51%, corresponding to improvements of 2.07 and 1.70 percentage points, respectively. The consistent improvements in both boundary-sensitive metrics further demonstrate that GCSA enhances the agreement between predicted and ground-truth change boundaries, providing direct quantitative support for its effectiveness in boundary localization. Therefore, after introducing GCSA, the proposed model not only reduces false positives and false negatives and improves the F1 score but also enhances changed-region localization accuracy, thereby improving the IoU. In terms of the magnitude of improvement, the GCSA module delivers consistent gains on both datasets, with more pronounced region-level improvements on the SYSU-CD dataset, which contains more complex scenes. It should be noted that these larger improvements on SYSU-CD refer to the region-level F1 and IoU metrics, whereas the boundary-sensitive BF1 and BIoU metrics exhibit larger gains on WHU-CD. This difference indicates that the contribution of GCSA is reflected differently across the two datasets: its boundary-refinement effect is more pronounced for the relatively distinct building contours in WHU-CD, while on the more complex SYSU-CD dataset, its contribution is more strongly reflected in the overall discrimination and localization of changed regions. In terms of absolute accuracy, all ablation configurations achieved higher F1 and IoU scores on the WHU-CD dataset than on the SYSU-CD dataset. This is primarily because the SYSU-CD dataset contains a greater variety of change types and more complex background interference, placing higher demands on the model’s generalization ability and its capacity to accurately identify changed regions. In terms of relative performance improvements, compared with the original STENet, the complete model achieved increases of 5.51 percentage points in F1 and 9.36 percentage points in IoU on the WHU-CD dataset and increases of 5.28 and 7.38 percentage points, respectively, on the SYSU-CD dataset. These results demonstrate that the dynamically weighted loss, SAM3 feature encoder, and GCSA module exhibit strong synergy, consistently improving performance in both building-change detection and complex land-cover change scenarios, thus confirming the effectiveness and robustness of the proposed method.
(4) Heatmap visualization analysis: Figure 6 and Figure 7 show the ablation feature visualization results of the proposed method on the WHU-CD and SYSU-CD datasets, respectively. As observed from the figures, the highlighted regions of the baseline method are relatively scattered, and some responses fall outside the real changed regions. This indicates that relying only on the original STENet features makes it difficult for the model to accurately focus on changed targets, and the model is easily affected by background textures and areas of pseudo-change. After introducing dynamic weights into our method, the model’s response brightness in regions of change increased, indicating that dynamic weights effectively improved the accuracy of detecting changed objects, while the response in regions without change decreased significantly. On this basis, after introducing SAM3 multiscale feature encoding, the model’s highlighted regions were significantly concentrated in the actual regions of change, while the irrelevant responses in the background regions were further attenuated. This suggests that the deep semantic features supplied by SAM3 improve the model’s discriminative capability for changed regions, thereby aligning the feature responses more closely with the true label regions. By further incorporating the GCSA attention mechanism, false positives in pseudo-change regions such as shadows and lighting variations are almost completely eliminated. At the same time, the edge responses in the feature maps of changed regions are enhanced, and the areas of focus in the heatmap largely coincide with the white changed regions in the ground-truth labels, resulting in clearer and more accurate boundary localization.

## 5. Conclusions

To enhance the feature representation of multi-scale change targets, change region boundary localization, and change region learning ability under class imbalance conditions in high-resolution optical remote sensing images, this study proposes a change detection network SAM3TGNet based on SAM3 feature encoding and a global contextual spatial attention mechanism. This model employs a parameter-frozen SAM3 visual encoder to extract hierarchical features from bi-temporal images and selects the last three layers of high-level semantic features as multi-scale spatial representations. After fusing the spatiotemporal features, the GCSA module enhances the consistency and continuity of the responses within the changing areas through global context modeling, while the local detail enhancement branch supplements boundary and fine structure information. The dynamic weighted loss adaptively adjusts the changed-class weight according to the proportion of changed pixels in each batch, thereby alleviating the class imbalance between changed and unchanged pixels. Experiments on the WHU-CD and SYSU-CD datasets showed that SAM3TGNet achieved mean F1 scores of 94.08 ± 0.12% and 82.91 ± 0.20%, and mean IoU scores of 88.82 ± 0.22% and 70.81 ± 0.29%, respectively, over three independent runs. It outperforms several comparison methods, demonstrating good detection performance in both building change detection and complex land-cover change scenarios. Although SAM3TGNet achieves promising results, the SAM3 visual encoder has a relatively large parameter scale, and the model has currently been validated on only two public datasets. Therefore, its inference efficiency and cross-scenario generalization capability still require further improvement. Future work will investigate more efficient adaptation strategies for the SAM3 encoder, including a systematic comparison of frozen, parameter-efficient fine-tuning, and full fine-tuning settings, to examine their trade-offs in feature adaptation, trainable parameter scale, and computational efficiency. In addition, alternative lightweight channel adaptation structures will be further investigated to evaluate their effects on multiscale feature alignment and computational efficiency. The feature fusion structure and attention mechanism will also be further optimized to improve the model’s efficiency and generalization capability. Although the results of three independent runs demonstrate good experimental stability, a systematic hyperparameter sensitivity analysis of the Top-(k_s) routing configuration in the cross-temporal attention module and the clipping range of the dynamic positive-class weight has not yet been conducted. Future work will further evaluate the effects of different parameter settings on detection accuracy and computational cost to provide a more comprehensive basis for parameter selection.

## Figures and Tables

**Table 1:** Confusion Matrix.

Ground Truth	Prediction	
	Positive	Negative
**Positive**	TP (True Positive)	FN (False Negative)
**Negative**	FP (False Positive)	TN (True Negative)

**Table 2:** Quantitative accuracy comparison of different change detection methods on the WHU-CD dataset. Bold values indicate the best result for each metric.

Method	Feature Encoder	WHU-CD			
		Pre (%)	Rec (%)	F1 (%)	IoU (%)
USSFCNet [22]	CNN	90.39	93.53	91.94	85.08
DFMANet [47]	CNN-Transformer	94.01	91.24	92.60	86.23
MPNet [48]	CNN	84.93	93.72	89.11	80.36
HANet [24]	CNN	92.14	90.52	91.32	84.03
STENet [41]	CNN	88.27	89.02	88.65	79.60
OUR	SAM3	**94.14 ± 0.13**	**94.02 ± 0.15**	**94.08 ± 0.12**	**88.82 ± 0.22**

**Table 3:** Quantitative accuracy comparison of different change detection methods on the SYSU-CD dataset. Bold values indicate the best result for each metric.

Method	Feature Encoder	SYSU-CD			
		Pre (%)	Rec (%)	F1 (%)	IoU (%)
USSFCNet [22]	CNN	78.18	81.07	79.60	66.11
DFMANet [47]	CNN-Transformer	**83.57**	82.15	82.86	70.73
MPNet [48]	CNN	80.72	82.97	81.83	69.25
HANet [24]	CNN	78.71	76.14	77.41	63.14
STENet [41]	CNN	78.51	76.80	77.65	63.46
OUR	SAM3	82.75 ± 0.23	**83.06 ± 0.17**	**82.91 ± 0.20**	**70.81 ± 0.29**

**Table 4:** Comparison of model parameters and computational efficiency among different change detection methods. Bold values indicate the results obtained using the complete SAM3TGNet model.

Methods	Trainable Params(M)	Frozen Params (M)	FPS (frame/s)	FLOPs (G)
USSFCNet [22]	**1.52**	0	82.75	**4.86**
DFMANet [47]	8.40	0	72.13	12.35
MPNet [48]	18.76	0	108.09	10.11
HANet [24]	2.61	0	186.56	17.67
STENet [41]	5.42	0	**263.09**	32.96
OUR	7.03	**449.01**	32.49	362.90

**Table 5:** Ablation results of the proposed method. Bold values indicate the best result for each metric.

Dataset	Method	Baseline	Dynamic Weighted Loss	SAM3 Encoder	GCSA	F1% (△)	IoU% (△)
WHU-CD	Variant1	√				88.65	79.60
	Variant2	√	√			91.73 (+3.08)	84.72 (+5.12)
	Variant3	√	√	√		92.95 (+4.30)	86.82 (+7.22)
	SAM3TGNet	√	√	√	√	**94.16 (+5.51)**	**88.96 (+9.36)**
SYSU-CD	Variant1	√				77.65	63.46
	Variant2	√	√			78.45 (+0.80)	64.54 (+1.08)
	Variant3	√	√	√		80.78 (+3.13)	67.75 (+4.29)
	SAM3TGNet	√	√	√	√	**82.93 (+5.28)**	**70.84 (+7.38)**

**Table 6:** Comparison of different loss functions on the WHU-CD and SYSU-CD datasets. Bold values indicate the results obtained using our proposed dynamically weighted BCE+Dice loss.

Dataset	Loss Function	F1% (△)	IoU% (△)
WHU-CD	BCE	88.65	79.60
	BCE + Dice	90.56 (+1.91)	82.75 (+3.15)
	Dynamic Weighted BCE + Dice (Ours)	**91.73 (+3.08)**	**84.72 (+5.12)**
SYSU-CD	BCE	77.65	63.46
	BCE + Dice	78.04 (+0.39)	63.99 (+0.53)
	Dynamic Weighted BCE + Dice (Ours)	**78.45 (+0.80)**	**64.54 (+1.08)**

**Table 7:** Boundary-sensitive evaluation of the GCSA module. “√” indicates that the GCSA module is introduced into the model. Bold values indicate the results obtained after incorporating the GCSA module.

Dataset	GCSA	BF1% (△)	BIoU% (△)
WHU-CD		81.68	65.49
	√	**87.51 (+5.83)**	**71.67 (+6.18)**
SYSU-CD		48.47	35.81
	√	**50.54 (+2.07)**	**37.51 (+1.7)**

## Data Availability

Publicly available datasets were analyzed in this study. The WHU-CD dataset is available at https://gpcv.whu.edu.cn/data/building_dataset.html (accessed on 20 July 2024). The SYSU-CD dataset is available at https://github.com/liumency/SYSU-CD (accessed on 29 June 2021). The source code repository for this study is available at https://github.com/zjy-0206/SAM3TGNet (accessed on 13 July 2026).

## References

[1] Xiong J., Liu F., Wang X., Yang C. (2024). Siamese Transformer-Based Building Change Detection in Remote Sensing Images. Sensors.

[2] Amini S., Saber M., Rabiei-Dastjerdi H., Homayouni S. (2022). Urban Land Use and Land Cover Change Analysis Using Random Forest Classification of Landsat Time Series. Remote Sens..

[3] Singh D.K., Hoskere V. (2023). Post Disaster Damage Assessment Using Ultra-High-Resolution Aerial Imagery with Semi-Supervised Transformers. Sensors.

[4] Wen D., Huang X., Bovolo F., Li J., Ke X., Zhang A., Benediktsson J.A. (2021). Change Detection from Very-High-Spatial-Resolution Optical Remote Sensing Images: Methods, Applications, and Future Directions. IEEE Geosci. Remote Sens. Mag..

[5] Wang Q., Li M., Li G., Zhang J., Yan S., Chen Z., Zhang X., Chen G. (2023). High-Resolution Remote Sensing Image Change Detection Method Based on Improved Siamese U-Net. Remote Sens..

[6] Li L., Li X., Zhang Y., Wang L., Ying G. Change Detection for High-Resolution Remote Sensing Imagery Using Object-Oriented Change Vector Analysis Method. Proceedings of the 2016 IEEE International Geoscience and Remote Sensing Symposium (IGARSS).

[7] Kılıç D.K., Nielsen P. (2022). Comparative Analyses of Unsupervised PCA K-Means Change Detection Algorithm from the Viewpoint of Follow-Up Plan. Sensors.

[8] Phiri D., Simwanda M., Nyirenda V., Murayama Y., Ranagalage M. (2020). Decision Tree Algorithms for Developing Rulesets for Object-Based Land Cover Classification. ISPRS Int. J. Geo-Inf..

[9] Shree N.G., Meiaraj C. (2025). Land Use and Land Cover Change Detection by Machine Learning Classifiers (SVM and RF) Using Satellite Remote Sensing Observations for Cuddalore Taluk, Tamil Nadu, India. J. Earth Syst. Sci..

[10] Bill Donatien L.M., Clobite B.B., Meris Midel M.L. (2024). Land Use Land Cover Change Detection Using Multi-Temporal Landsat Imagery in the North of Congo Republic: A Case Study in Sangha Region. Geocarto Int..

[11] Shi W., Zhang M., Zhang R., Chen S., Zhan Z. (2020). Change Detection Based on Artificial Intelligence: State-of-the-Art and Challenges. Remote Sens..

[12] Li J., Cai Y., Li Q., Kou M., Zhang T. (2024). A Review of Remote Sensing Image Segmentation by Deep Learning Methods. Int. J. Digit. Earth.

[13] Daudt R.C., Le Saux B., Boulch A. Fully Convolutional Siamese Networks for Change Detection. Proceedings of the 2018 25th IEEE International Conference on Image Processing (ICIP).

[14] Fang S., Li K., Shao J., Li Z. (2022). SNUNet-CD: A Densely Connected Siamese Network for Change Detection of VHR Images. IEEE Geosci. Remote Sens. Lett..

[15] Chen H., Qi Z., Shi Z. (2022). Remote Sensing Image Change Detection with Transformers. IEEE Trans. Geosci. Remote Sens..

[16] Kirillov A., Mintun E., Ravi N., Mao H., Rolland C., Gustafson L., Xiao T., Whitehead S., Berg A.C., Lo W.-Y. Segment Anything. Proceedings of the 2023 IEEE/CVF International Conference on Computer Vision (ICCV).

[17] Carion N., Gustafson L., Hu Y.-T., Debnath S., Hu R., Suris D., Ryali C., Alwala K.V., Khedr H., Huang A. (2025). SAM 3: Segment Anything with Concepts. arXiv.

[18] Chen W., Ouyang S., Tong W., Li X., Zheng X., Wang L. (2022). GCSANet: A Global Context Spatial Attention Deep Learning Network for Remote Sensing Scene Classification. IEEE J. Sel. Top. Appl. Earth Obs. Remote Sens..

[19] Ho Y., Wookey S. (2020). The Real-World-Weight Cross-Entropy Loss Function: Modeling the Costs of Mislabeling. IEEE Access.

[20] Sudre C.H., Li W., Vercauteren T., Ourselin S., Cardoso M.J. (2017). Generalised Dice Overlap as a Deep Learning Loss Function for Highly Unbalanced Segmentations. Deep Learning in Medical Image Analysis and Multimodal Learning for Clinical Decision Support.

[21] Song K., Jiang J. (2021). AGCDetNet: An Attention-Guided Network for Building Change Detection in High-Resolution Remote Sensing Images. IEEE J. Sel. Top. Appl. Earth Obs. Remote Sens..

[22] Lei T., Geng X., Ning H., Lv Z., Gong M., Jin Y., Nandi A.K. (2023). Ultralightweight Spatial–Spectral Feature Cooperation Network for Change Detection in Remote Sensing Images. IEEE Trans. Geosci. Remote Sens..

[23] Chen H., Shi Z. (2020). A Spatial-Temporal Attention-Based Method and a New Dataset for Remote Sensing Image Change Detection. Remote Sens..

[24] Han C., Wu C., Guo H., Hu M., Chen H. (2023). HANet: A Hierarchical Attention Network for Change Detection with Bitemporal Very-High-Resolution Remote Sensing Images. IEEE J. Sel. Top. Appl. Earth Obs. Remote Sens..

[25] Radford A., Kim J.W., Hallacy C., Ramesh A., Goh G., Agarwal S., Sastry G., Askell A., Mishkin P., Clark J. (2021). Learning Transferable Visual Models from Natural Language Supervision. Machine Learning Research, Proceedings of the 38th International Conference on Machine Learning, Virtual, 18–24 July 2021.

[26] Dong S., Wang L., Du B., Meng X. (2024). ChangeCLIP: Remote Sensing Change Detection with Multimodal Vision-Language Representation Learning. ISPRS J. Photogramm. Remote Sens..

[27] Li K., Cao X., Meng D. (2024). A New Learning Paradigm for Foundation Model-Based Remote-Sensing Change Detection. IEEE Trans. Geosci. Remote Sens..

[28] Siméoni O., Vo H.V., Seitzer M., Baldassarre F., Oquab M., Jose C., Khalidov V., Szafraniec M., Yi S., Ramamonjisoa M. (2025). DINOv3. arXiv.

[29] Dong S., Hu Y., Wang L., Chen G., Meng X. (2026). PeftCD: Leveraging Vision Foundation Models with Parameter-Efficient Fine-Tuning for Remote Sensing Change Detection. IEEE J. Sel. Top. Appl. Earth Obs. Remote Sens..

[30] Cheng C.-H., Hsu C.C. (2026). ChangeDINO: DINOv3-Driven Building Change Detection in Optical Remote Sensing Imagery. ISPRS Ann. Photogramm. Remote Sens. Spat. Inf. Sci..

[31] Ding L., Zhu K., Peng D., Tang H., Yang K., Bruzzone L. (2024). Adapting Segment Anything Model for Change Detection in VHR Remote Sensing Images. IEEE Trans. Geosci. Remote Sens..

[32] Gao J., Zhang D., Wang F., Ning L., Zhao Z., Li X. (2025). Combining SAM with Limited Data for Change Detection in Remote Sensing. IEEE Trans. Geosci. Remote Sens..

[33] Zhao X., Wu Z., Chen Y., Zhou W., Wei M. (2024). Fine-Grained High-Resolution Remote Sensing Image Change Detection by SAM-UNet Change Detection Model. Remote Sens..

[34] Zhang D., Wang F., Ning L., Zhao Z., Gao J., Li X. (2024). Integrating SAM with Feature Interaction for Remote Sensing Change Detection. IEEE Trans. Geosci. Remote Sens..

[35] Wei C., Wu X., Wang B. (2025). ASS-CD: Adapting Segment Anything Model and Swin-Transformer for Change Detection in Remote Sensing Images. Remote Sens..

[36] Liu S., Zhao D., Tang L. (2026). A Multi-Scale Remote Sensing Image Change Detection Network Based on Vision Foundation Model. Remote Sens..

[37] Zhang M., Hu J., Li Q., Wang Q. (2026). Exploring Generalizable Remote Sensing Change Detection via Low-Rank Exchange Adaptation of Vision Foundation Model. Proc. AAAI Conf. Artif. Intell..

[38] Wang J., Yu Q., Zhang R., Xiao N. (2026). TriFusion-CD: Tri-Source Fusion for Robust Remote Sensing Change Detection Under Pseudo-Change Interference. Remote Sens..

[39] Shi Y., Yang R., Yin C., Lu Y., Huang B., Wen Y., Zhong Y., Gu Z. (2026). MV-S2CD: A Modality-Bridged Vision Foundation Model-Based Framework for Unsupervised Optical–SAR Change Detection. Remote Sens..

[40] Huo C., Chen K., Zhang S., Wang Z., Yan H., Shen J., Hong Y., Qi G., Fang H., Wang Z. (2025). When Remote Sensing Meets Foundation Model: A Survey and Beyond. Remote Sens..

[41] Pan X., Lai J., Jin Y., Zhou X., Zheng J. (2024). STENet: A Spatial Selection and Temporal Evolution Network for Change Detection in Remote Sensing Images. IEEE Trans. Geosci. Remote Sens..

[42] Zhu L., Wang X., Ke Z., Zhang W., Lau R.W.H. BiFormer: Vision Transformer with Bi-Level Routing Attention. Proceedings of the IEEE/CVF Conference on Computer Vision and Pattern Recognition (CVPR).

[43] Zamir S.W., Arora A., Khan S., Hayat M., Khan F.S., Yang M.-H. Restormer: Efficient Transformer for High-Resolution Image Restoration. Proceedings of the IEEE/CVF Conference on Computer Vision and Pattern Recognition (CVPR).

[44] Ji S., Wei S., Lu M. (2019). Fully Convolutional Networks for Multisource Building Extraction from an Open Aerial and Satellite Imagery Data Set. IEEE Trans. Geosci. Remote Sens..

[45] Shi Q., Liu M., Li S., Liu X., Wang F., Zhang L. (2022). A Deeply Supervised Attention Metric-Based Network and an Open Aerial Image Dataset for Remote Sensing Change Detection. IEEE Trans. Geosci. Remote Sens..

[46] Cheng B., Girshick R., Dollár P., Berg A.C., Kirillov A. Boundary IoU: Improving Object-Centric Image Segmentation Evaluation. Proceedings of the IEEE/CVF Conference on Computer Vision and Pattern Recognition (CVPR).

[47] Zhan T., Tian Q., Zhu Y., Lan J., Dang Q., Gong M. (2025). Difference-Aware Multiscale Feature Aggregation Network for Building Change Detection. IEEE Trans. Geosci. Remote Sens..

[48] Wei H., Liu Y., Ma Y., Pang D., Ye Y., Sui X., Chen Q. (2025). Multiscale Spatial Frequency Fusion and Prior Change Guidance Network for Remote Sensing Change Detection. IEEE Trans. Instrum. Meas..

[49] Zhu L. THOP: PyTorch-OpCounter. https://github.com/Lyken17/pytorch-OpCounter.

